# Combined MRI, high-resolution manometry and a randomised trial of bisacodyl versus hyoscine show the significance of an enlarged colon in constipation: the RECLAIM study

**DOI:** 10.1136/gutjnl-2024-332755

**Published:** 2024-10-22

**Authors:** Victoria Wilkinson-Smith, Mark Scott, Alex Menys, Lukasz Wiklendt, Luca Marciani, David Atkinson, Stefano Sansone, Ausra Zdanaviciene, Carol Coupland, Charles H Knowles, Philip Dinning, Stuart A Taylor, Penny Gowland, Caroline Louise Hoad, Maura Corsetti, Robin C Spiller

**Affiliations:** 1 School of Medicine, University of Nottingham, Nottingham, UK; 2 NIHR Nottingham Biomedical Research Centre, Nottingham, UK; 3 Academic Surgical Unit, Barts and The London School of Medicine and Dentistry, London, UK; 4 Motilent Ltd, London, UK; 5 College of Medicine & Public Health, Flinders University, Adelaide, South Australia, Australia; 6 Translational Medical Sciences, Nottingham Digestive Diseases Centre, University of Nottingham, Nottingham, UK; 7 Nottingham Digestive Diseases Centre, NIHR Nottingham Biomedical Research Centre, Nottingham, UK; 8 Centre for Medical Imaging, UCL, London, UK; 9 Nottingham Digestive Diseases Centre, Nottingham University Hospitals NHS Trust, Nottingham, UK; 10 Barts and the London School of Medicine and Dentistry, Queen Mary University, London, UK; 11 Centre for Academic Primary Care, University of Nottingham, Nottingham, UK; 12 Blizzard Institute, Queen Mary University, London, UK; 13 Gastroenterology and Surgery, Flinders Medical Center, Adelaide, South Australia, Australia; 14 Imaging Department, University College London Hospitals NHS Foundation Trust, London, UK; 15 Sir Peter Mansfield Magnetic Resonance Centre, University of Nottingham, Nottingham, UK; 16 Nottingham Digestive Diseases Biomedical Research Unit, University of Nottingham, Nottingham, UK; 17 Translational Medical Science, University of Nottingham, Nottingham, UK

**Keywords:** CONSTIPATION, COLORECTAL MOTILITY, COLORECTAL PHYSIOLOGY, LAXATIVES

## Abstract

**Background:**

Colonic motility in constipation can be assessed non-invasively using MRI.

**Objective:**

To compare MRI with high-resolution colonic manometry (HRCM) for predicting treatment response.

**Design:**

Part 1: 44 healthy volunteers (HVs), 43 patients with irritable bowel syndrome with constipation (IBS-C) and 37 with functional constipation (FC) completed stool diaries and questionnaires and underwent oral macrogol (500–1000 mL) challenge. Whole gut transit time (WGTT), segmental colonic volumes (CV), MRI-derived Motility Index and chyme movement by ‘tagging’ were assessed using MRI and time to defecation after macrogol recorded. Left colonic HRCM was recorded before and after a 700 kcal meal. Patients then proceeded to Part 2: a randomised cross-over study of 10-days bisacodyl 10 mg daily versus hyoscine 20 mg three times per day, assessing daily pain and constipation.

**Results:**

Part 1: Total CVs median (range) were significantly greater in IBS-C (776 (595–1033)) and FC (802 (633–951)) vs HV (645 (467–780)), p<0.001. Patients also had longer WGTT and delayed evacuation after macrogol. IBS-C patients showed significantly reduced tagging index and less propagated pressure wave (PPW) activity during HRCM versus HV. Compared with FC, IBS-C patients were more anxious and reported more pain. Abnormally large colons predicted significantly delayed evacuation after macrogol challenge (p<0.02), impaired manometric meal response and reduced pain with bisacodyl (p<0.05).

Part 2: Bisacodyl compared with hyoscine increased bowel movements but caused more pain in both groups (p<0.03).

**Conclusion:**

An abnormally large colon is an important feature in constipation which predicts impaired manometric response to feeding and treatment responses. HRCM shows that IBS-C patients have reduced PPW activity.

**Trial registration number:**

The study was preregistered on ClinicalTrials.gov, Reference: NCT03226145.

**WHAT IS ALREADY KNOWN ON THIS TOPIC**

- MRI can be used to assess colonic volumes and motor response after a macrogol challenge in patients with constipation.

**WHAT THIS STUDY ADDS**

- MRI-assessed colonic volumes are greater in both functional constipation (FC) and irritable bowel syndrome (IBS) patients than in healthy volunteers (HVs).
- Large colons (>90th centile for HVs) predict impaired manometric meal response, delayed evacuation after macrogol challenge and reduced pain with bisacodyl.
- Compared with FC, patients with IBS-C show reduced propagated pressure waves in the left colon and report more pain after macrogol and bisacodyl.

**HOW THIS STUDY MIGHT AFFECT RESEARCH, PRACTICE OR POLICY**

- MRI assessment of colonic volumes could contribute to individualised treatment of constipation in secondary/tertiary care.

## Introduction

 Constipation is a common symptom affecting approximately 11%–15% of the general population.^1 2^ The symptom-based Rome IV classification separates functional constipation (FC) from irritable bowel syndrome with constipation (IBS-C),^3^ but this subdivision is controversial^4^,^7^ as symptoms overlap substantially.^8^ Treatments targeting these different populations give a number needed to treat varying from 2 to 7,^9^ leaving many patients dissatisfied.^10^ Many investigators are attempting to improve this by more accurate assessment of the underlying pathophysiology which is recognised to comprise three principal overlapping factors: delayed transit secondary to gut dysmotility, evacuatory dysfunction and abnormal sensory function, which is often allied to an enlarged or hypercompliant bowel.^7^ Current diagnostic tests are based primarily on assessments of transit including scintigraphy and radio-opaque markers and only rarely manometry and barostat owing to the complexity of the latter techniques. By contrast, MRI, which has as yet not been widely used, offers the opportunity to assess many parameters simultaneously. Using MRI, we have developed an objective measure of colonic function, the macrogol challenge^11^ which measures colonic volumes (CVs) and maximal motor response (maximal MRI Motility Index, MMI) to macrogol^12^ and when combined with non-absorbable markers can reproducibly measure whole gut transit time (WGTT).^13^ While there are currently only a few studies using MRI, our pilot study suggested that compared with IBS-C, FC patients had larger CVs, longer transit and reduced motility response,^11^ as assessed by either MMI or a colon ‘tagging’ technique, a recognised measure of movement of colonic content.^14^ We hypothesised that MRI assessment of CV and motility would allow better targeting of treatment.

High-resolution colonic manometry (HRCM) provides new insights revealing co-ordinated and often retrograde-moving patterns of colonic contraction—‘the cyclic motor pattern’ (CMP) particularly in the sigmoid colon, suggesting a ‘brake’ function.^15^ Such recordings also demonstrate that patients with slow-transit constipation show a reduced colonic response to feeding,^16^ but it is unclear if this differs from patients with IBS-C.

The primary aims were therefore to (1) compare the non-invasive, patient-acceptable, MRI characterisation of colonic motor function in both FC and IBS-C against the more demanding and invasive HRCM and (2) test in a randomised, double-blinded, cross-over trial of the hypothesis that colonic motility, studied with MRI would predict the difference in response to a colonic motor stimulant (bisacodyl) compared with an antispasmodic (hyoscine butylbromide). The logic behind this comparison was the earlier findings that IBS patients had smaller colons which could have reflected increased colonic tone and motility. This would be expected to respond to an anti-spasmodic such as hyoscine with reduced symptoms, particularly pain. In contrast, the FC patients in the previous study had both larger colons and reduced motility both of which should have improved with a prokinetic. By using a common endpoint namely pain we aimed to assess a difference between treatments which could be correlated with our MRI measurements.

## Methods

We performed the study in two parts.

### Part 1: MRI and manometry

Participants with constipation and healthy volunteer (HV) were recruited at two sites in the UK (Nottingham and London) from both primary and secondary care (online supplemental file A). All participants underwent a 2-week screening period (off laxatives) during which a bowel habit diary including Bristol Stool Form Scale (BSFS) was completed, along with baseline Hospital Anxiety and Depression Scale (HADS) and the Patient Assessment of Constipation-Symptoms (PAC-SYM)^17^ score. A modified PAC-SYM (mPAC-SYM) score was calculated using only abdominal pain, discomfort and cramps elements of PAC-SYM. Subjects also underwent a balloon expulsion test to assess the ability to expel a rectal balloon (online supplemental file B1).

All participants attended fasted on two separate occasions for (1) a 2-hour MRI study and (2) a 4-hour HRCM study.

#### MRI study

Participants consumed 5 transit markers 24 hours before a fasting scan, then ingested oral macrogol provided as MoviPrep (10 mL/kg body weight, minimum 500 mL, maximum 1000 mL), followed by MRI scans at 60 and 120 min. 1 litre MoviPrep contains 100 g of polyethylene glycol (PEG) 3350, 7.5 g sodium sulfate, 2.69 g sodium chloride, 1.01 g of potassium chloride plus aspartame, acesulfame potassium and lemon flavouring, hereafter referred to as macrogol. Images were analysed blind to participant condition, by a single operator (VW-S) to assess total and segmental CVs, motility measures including ascending colon (AC) ‘tagging index’, AC and descending colon (DC) MMI at 60 and 120 min and WGTT (online supplemental C1,C2).

##### Primary endpoint

MMI of the AC derived from wall movement at maximum distension to macrogol as previously described.^18^

##### Secondary endpoints

CVs, peak MMI of the DC, WGTT, assessed by the ‘Weighted Average Position Score’ (WAPS) of transit markers as previously validated^13^ and time to first bowel movement following macrogol.

##### Exploratory endpoints

Movement of AC colonic chyme as assessed from the ‘tagging index’^19^ (online supplemental C2), pain scores 0–2 hours after macrogol (0–3 scale).

### HRCM study

Participants received a tap water enema to cleanse the left colon prior to flexible sigmoidoscopy and placement of the HRCM catheter.^20^ Recordings were performed for 2 hours before and after a 700 kcal meal. Using previously developed software (PlotHRM),^15^ manometric traces were examined for the presence of the CMP and high-amplitude propagating contractions (HAPCs) in the hour before and after the meal. Further automated analysis of other motor patterns activity including propagated pressure waves (PPW) was then performed using a Bayesian functional mixed-effects model^21 22^ (online supplemental D2.1, figure S5).

#### Primary endpoint

Percentage time occupied by the CMP (CMP in the sigmoid colon following the meal.

#### Secondary endpoints

HAPCs per hour and measures of coordinated antegrade and retrograde propagated contractions (analysis detailed in online supplemental D2.1).

### Part 2: randomised, placebo-controlled trial comparing bisacodyl and hyoscine

#### Study design

Constipated subjects from part 1 were invited to take part in a randomised double-blind, double-dummy, cross-over study comparing bowel habit and pain response to a 10-day treatment with either a stimulant laxative, bisacodyl (10 mg daily) or a muscle relaxant, hyoscine butylbromide (20 mg three times per day). Active drug and placebo were provided as identical-appearing overencapsulated capsules, one taken three times daily and one once a day.

Concealed allocation was performed using a numbered container with the sequence bisacodyl versus hyoscine being randomly allocated by Nottingham hospital pharmacy who kept the code, which was not released until data lock. Participants completed a daily diary documenting the number of bowel movements and for each bowel movement, the BSFS and feeling of completeness of evacuation. Each day, they also recorded a pain score (in answer to the question what their ‘worst’ pain was in that 24-hour period, scored from 1 to 5) and completed a modified mPAC-SYM questionnaire (online supplemental E1.1) before and after the treatment period. Rescue medication (prucalopride, senna or sodium picosulphate based on what they had used before) was allowed if they had no bowel movement for 3 days. Dose reduction was permitted for excessive side effects (see online supplemental E.1). Data were collected on paper CRFs and diaries and collated with both participants and investigators blinded to active ingredient. Unblinding was performed only after completion of data collection and data lock.

##### Primary endpoint

Difference in average worst daily pain between bisacodyl and hyoscine intervention periods.

##### Secondary endpoints

The number of complete spontaneous bowel movements (CSBM), mPAC-SYM score and number of days with either hard (BSFS 1 or 2) or no stool.

##### Exploratory endpoints

We also determined whether any objective MRI or manometry measures could predict clinical response as defined by other authors. A bisacodyl ‘responder’ was defined as a patient who had an increase in 1 CSBM per week^23^ while hyoscine ‘responder’ had a reduction in (m) PAC-SYM by the previously defined minimal clinically important difference of >0.6 points (ie, reduction in pain).^24^

### Statistical analysis

Basic characteristics of the study population, as well as the MRI, HRCM, clinical trial and symptom data, were summarised using frequencies, percentages, means and SD or medians with IQRs as appropriate to the distribution.

Differences between participant groups for continuous variables were assessed using either analysis of variance (ANOVA) or mixed effect models with Tukey’s multiple comparisons test for post hoc comparisons between groups and χ^2^ tests for categorical data. Comparisons between FC and IBS-C were done using an unpaired t-test or Mann-Whitney U test depending on the distribution of the data. The difference in pain scores between baseline and trial period was analysed separately for each drug (using a Student’s t-test if normally distributed or Mann- Whitney U test if not) comparing those with baseline volume >90th centile with those with normal volumes.

Correlations were assessed using the Pearson correlation coefficient for normally distributed data or the Spearman correlation coefficient for non-normally distributed data. Statistical tests were performed using GraphPad Prism V.9 for Windows (GraphPad Software, La Jolla, California, USA).

### Sample size considerations

#### Part 1

Primary objective: We aimed for a level of agreement between MRI and manometry >70% which we could estimate to be within ±10% (95% CI) using 80 patients, assuming a proportion of 0.5 in each group (hypomotile vs normal/hypermotile).

#### Part 2

There are no previous data on which to base a power calculation so we invited all patients from study 1.

## Results part 1

### Clinical characteristics

We enrolled 44 HV, 43 participants with IBS-C and 38 with FC of whom 121 completed part 1 and 72 participated in part 2 (Consort diagram in online supplemental figure S1). Participants were predominantly middle-aged females (116/125) though HVs were younger than the patients (table 1). Participants in all three groups reported similar numbers of attempted bowel movements in the 14-day diary, however, the FC group had fewer spontaneous bowel movements (SBMs) (table 1, online supplemental B3 table S1). Both patient groups with constipation had fewer CSBM and harder stools on the BSFS than HVs. Modified PAC-SYM (mPAC-SYM) scores (see online supplemental E1.1) were significantly higher (indicating worse symptoms of pain, discomfort and cramps) in the IBS-C group compared with FC, both being considerably higher than HVs (table 1). Both patient groups had significantly higher depression scores than HVs with IBS-C patients also having significantly higher anxiety scores. A rectal balloon was expelled in the defined time by 89% of HV, 84% IBS-C and 75% FC (p=0.27) (online supplemental B1).

**Table 1:** Demographics, baseline stool diary and psychological depression and anxiety scores

	HV (n=44)	IBS-C (n=43)	FC (n=38)	P value
Age	33±12	40±13*	46±14*	<0.001
Gender (female %)	39 (89)	41 (95)	36 (95)	0.41
BMI	25±5	26±5	25±5	0.98
Screening stool diary (14-day diary)				
Total BM attempts	17±7	16±13	18±18	0.74
Number of SBM	17±6	14±13	10±11*	0.18
Number of CSBM	15±7	1±2*	2±3*	<0.01
Average BSFS*	4±1	2±1*	2±4*	<0.01
mPAC-SYM	0.2±0.3	2.2±0.8*	1.2±0.9*†	<0.001
Psychological distress (median (IQR))				
HAD Score anxiety	5 (2–8)	7 (4.5–11)*	5 (2.3–7)	0.04
HAD Score depression	1 (0–2.3)	4 (1.5–8)**	2 (1–7)	0.0001

Comparison between groups performed using ANOVA, followed by Tukey’s Mmultiple Ccomparisons, apart from sex and % passing balloon expulsion test, which were analysed using the Chi-squaredχ2 test. * p<0.05 vs HVs, ** p<0.001 vs HV, † p<0.05 vs IBS* p HVs, ** p HV, † p IBS

Data presented as mean±SD except HADs.

* 2 week average of daily average stool form, excluding any post rescue therapy.

ANOVAanalysis of varianceBMIbody mass indexBSFSBristol Stool Form ScaleCSBMcomplete spontaneous bowel movementsHADsHospital Anxiety and Depression ScaleHVshealthy volunteersIBSirritable bowel syndromemPAC-SYMmodified Patient Assessment of Constipation-Symptoms

### MRI outcomes

#### Baseline colon volumes

FC and IBS-C patients had significantly larger mean total baseline CVs than HVs, this difference (approximately 20% increase) being mainly due to increased transverse colon (TC) volumes (TCVs) in both IBS-C and FC with a significant increase in AC volume in IBS-C while DC and rectosigmoid colon (RS) volumes did not differ from HVs (table 2). There were no correlations between baseline CVs and MMI, tagging index or WGTT (online supplemental F2 table S2).

**Table 2:** Total and segmental volumes median (IQR)

Group	n	Ascending colon	Transverse colon	Descending colon	Rectosigmoid	Total CV
HVs	41	205 (143–265)	198 (139–253)	119 (67–163)	106 (64–158)	645 (467–780)
IBS-C	43	261 (203–298)*	292 (187–377)**	119 (73–168)	109 (78–145)	776 (595–1033)**
FC	36	227 (195–292)	313 (198–420)**	107 (76–168)	121 (84–174)	802 (633–951)**

Two-way repeated measures ANOVA showed significant effect of group F=736, p<0.001 and segment F=7.2, p<0.001 and interaction F=6.3, p<0.001.

* p<0.05 vs HV. **p<0.01 vs HV.

ANOVAanalysis of varianceCVcolonic volumeFCfunctional constipationHVshealthy volunteersIBS-Cirritable bowel syndrome with constipation

25 patients with constipation had total CVs exceeding the 90th centile of HVs (923 mL). This increase in CVs was seen equally in all four segments (online supplemental F3 table S3). These patients were equally distributed between IBS-C and FC with no significant difference in age or HADS. However, such patients did tend to have reduced tagging index, harder stools (p=0.06) and slower transit but this was not significant, p=0.2 (table 3).

**Table 3:** Comparison of patients with enlarged colon versus normal-sized colon

	n	IBS-C/FC	TCV mL	Tagging index	Transit hours	CSBM/ wk	BSFS
Enlarged colon	25	15/10	1094(996–1244)	20.9(6.0)	84 (48)	0.0(0–1)	1.1 (1.2)
Normal-sized colon	54	28/26	707 (547–793)	25.1(9.7)	66 (61)	1 (0–3)	2.1 (1.3)
P value		0.62*	<0.0001†	0.06†	0.2†	0.2‡	‡0.06

* Fisher’s exact test.

† T-test.

‡ Mann-Whitney U test.

BSFSBristol Stool Form ScoreCSBMcomplete spontaneous bowel movementsFCfunctional constipationIBS-Cirritable bowel syndrome with constipationTCVtransverse colon volume

#### Effect of macrogol challenge on MRI outcomes, time to first bowel movement and pain

##### MMI Motility Index

**Figure 1:**
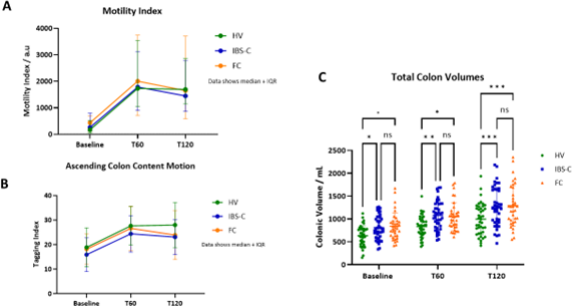
MRI Motility Index (MMI), ascending Colon Content Movement and Total Colonic Volumes. (A) Ascending colon MMI This rose significantly over time (p<0.001) ANOVA showed effect of time p<0.001, effect of group NS, p=0.97. (B) Ascending colon content motion was assessed by Tagging index at baseline and 60 (T60) and 120 min (T120) after macrogol ingestion. Tagging index showed a significant increase over time, which was less than HV in IBS-C (*p=0.02) but not in FC (p=0.08) at 120 min (two-way ANOVA, Tukey’s MC.) (C) Total colonic volumes. These rose over time for all groups. Both FC and IBS-C total colonic volumes were greater than HVs but not different from each other two-way ANOVA, Time effect p<0.0001, group effect p=0.0019, post hoc comparisons using Tukey’s multiple comparisons *p<0.05, **p<0.01, ***p<0.001 vs HV. ANOVA, analysis of variance; FC, functional constipation; HV, healthy volunteer; IBS-C, irritable bowel syndrome with constipation; NS, not significant.

Our primary endpoint, the MMI rose significantly from baseline in all three groups. MMI at T60 median (IQR) for HV was 1732 (1060–3535), 1785 (897–3125) for IBS-C and 2004 (713–3742) for FC. As figure 1A shows there was wide individual variability with no differences between groups. This was also true for the DC (online supplemental figure S6).

##### Tagging index

Our other measure of motility, the ‘tagging index’ reflecting movement of colonic chyme was also significantly increased after macrogol in all three groups at 60 and 120 min. However, this index was lower in IBS-C, significantly so at 120 min compared with HVs which did not differ from FC patients (figure 1B).

##### Colonic volumes

TCVs rose significantly after macrogol (T60 and T120) for all three groups with significant difference between HV and patients (FC and IBS-C) who showed substantial overlap (figure 1C).

##### Pain after macrogol challenge

**Figure 2:**
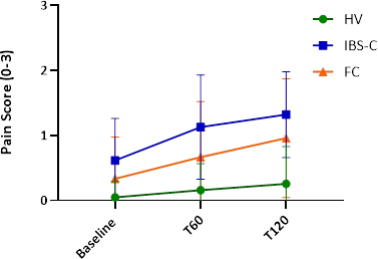
Pain on MRI study day pain score (0–3) is shown at baseline, 60 (T60) and 120 (T120) minutes after macrogol^R^ ingestion. IBS-C and FC had significantly more pain than HVs, p<0.05 at all 3 time points and at T60 IBS-C>FC, p<0.05. Mixed effect model (Restricted maximum likelihood) with Tukey’s MC. FC, functional constipation; HVs, healthy volunteers; IBS-C, irritable bowel syndrome with constipation.

Overall, both patient groups reported more pain than the HVs following ingestion of macrogol with greater pain at 60 min for IBS-C compared with FC (figure 2 and online supplemental F4 table S4). The reporting of pain was associated with a significantly higher peak volume 1277 (345) vs 1126 (410) ml but there was a wide scatter, p=0.04, (unpaired t-test) (online supplemental figure S7).

##### Time to bowel movement after macrogol

This was used to assess overall colonic responsiveness. While most (30/42, 70%) HV had a bowel movement <150 min following macrogol this was only true in 19/40 (47.5%) IBS-C and 14/32 (43.8%) FC (online supplemental figure S8), χ^2^ test p=0.028). Patients with abnormal enlarged colons had significantly delayed time to evacuate after macrogol, median (IQR) 180 (118–236), n=22 (3 failed to record) compared with the remaining patients 134 (89–180), n=50 p=0.02 Mann-Whitney U test. When we separately analysed the patients by Rome classification subgroups, two-way ANOVA showed no difference between IBS-C and FC (p=0.88) but a significant effect of enlarged colon, p=0.02.

##### Whole gut transit

WAPS showed substantial variability but was significantly higher in patients compared with HVs, with a median (IQR) score of 2.2 (0.6–3.4) vs 1 (0–2.3), respectively; p=0.03 (online supplemental figure S9). These scores are equivalent to a WGTT of 69 (21–104) vs 34 (4–69) hours if using the radio-opaque marker technique.^13^ However, there was no significant difference between IBS-C and FC patient groups (WAPS median 2.3 (IQR 0.6–4) and 1.7 (0.2–3.1), respectively, p=0.6).

### High-resolution colonic manometry

**Figure 3:**
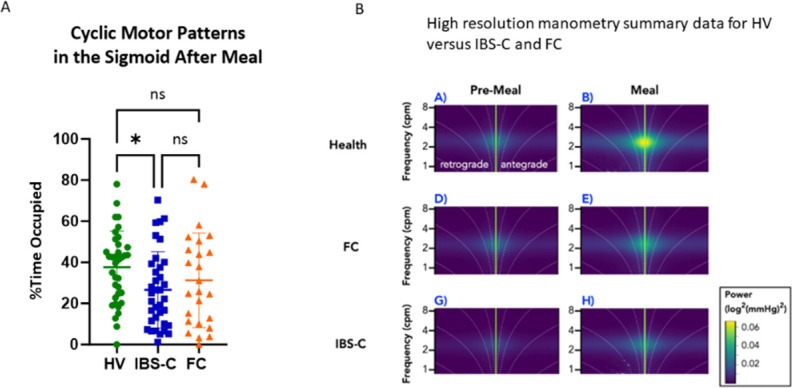
High-resolution colonic manometry before and after meal in HV, IBS-C and FC. (A) Cyclic motor patterns (CMPs) in the sigmoid colon after meal. The percentage of time occupied by the CMPs in the sigmoid colon 1 hour after the meal was significantly lower in IBS-C (27 (SD 19)%) but not FC group (38 (SD 18)%) compared with HVs (38 (18)), * = p=0.049, ANOVA with Tukey’s MCs. (B) High-resolution manometry summary data for propagated PWs in HV versus IBS-C and FC. This shows premeal and postmeal frequency distribution on the vertical axis and phase on the horizontal axis. Points to the right of 0 indicate antegrade propagated waves while those to the left indicate retrograde. Higher power is indicated by yellow showing IBS patients had significantly less power than HV or FC, both premeal and postmeal (for full analysis see online supplemental D2 and G2). ANOVA, analysis of variance; FC, functional constipation; HV, healthy volunteer; IBS-C, irritable bowel syndrome with constipation; PW, pressure wave.

HRCM data were obtained from 97 participants (36 HVs, 36 IBS-C and 25 FC). The CMP was observed in the sigmoid colon in the majority of participants both before and after the meal: 35/36 HVs, 36/36 IBS-C and 24/25 FC. The percentage of time occupied by the CMP in the sigmoid colon following the meal showed wide variability but was significantly lower in the IBS-C but not FC group compared with HVs (figure 3A).

HAPCs were identified in only a minority of participants both before and after the test meal: HV 6 and 8/36; IBS-C 4 and 6/36; FC 1 and 5/25, respectively. Due to the low number of subjects with HAPCs, statistical comparisons were not performed.

The meal induced an increase in the power of pressure waves (PW) in all three groups, an effect which did not differ significantly between the groups (online supplemental figure S10). However, when looking at the coordination of PW into propagating PW (PPW)s, significant differences emerged. During both the baseline and postprandial period, the power of PPWs was reduced in the IBS-C group compared with both HV and FC (figure 3B, online supplemental figure S11).

### Impact of enlarged colon volume on manometric features

**Figure 4:**
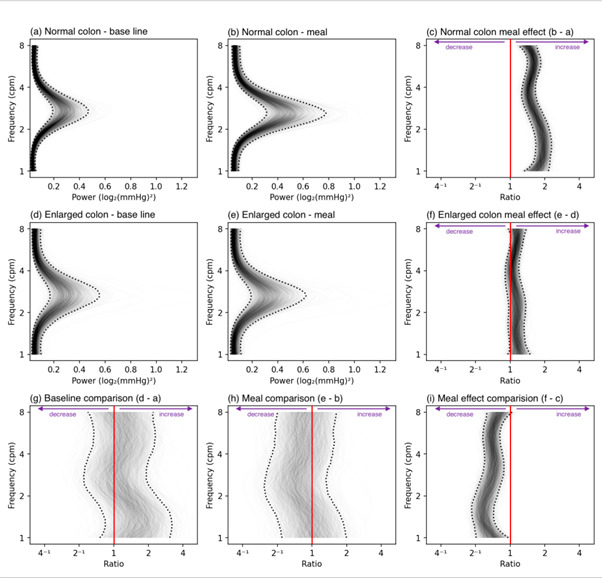
One-dimensional (1D) analysis of pressure waves (PW) at frequencies between 1 and 8 cycles per minute (cpm) in the sigmoid colon. Patients with a normal volume colon (top row; **a–c**) and patients with an enlarged volume colon (middle row; **d–f**) left column (**a, d, g**) shows baseline and middle column (**b, e, h**) the meal periods. In each image, the frequency of PW is shown on the y-axis. In (**a, b, d, e**) power is shown on the x-axis. The power refers to the prevalence of the PW at any of the calculated frequencies. 2000 overlapping grey lines in each panel represent posterior samples, and the dotted black lines form envelopes of 95% credible intervals. (**g**, **h**) The power ratio across the frequency range, between the enlarged and normal colons. When the entire envelope lies to one side of the vertical red line (which represents a ratio of 1), this shows a significant deviation (to the left a decrease in PWs in the enlarge compared with normal colons; to the right of the red line indicates an increase in PWs). During baseline and a meal period the red lines in (**g, h**) lie entirely within the grey envelope indicating no significant difference. **(c, f**) The ratio of the power of baseline activity to meal activity. In the normal colons (**c**), the grey envelope lies to the right of the red line indicating that the meal induced a significant increase in power in frequencies between 1 and 8 cpm. In patients with an enlarged colon (**f**), no meal response is seen (red line lies within the grey envelope). (**i**) The meal effect between the two groups, as the grey envelope lies to the left of the red line indicates that patients with an enlarged colon have a significantly reduced meal response compared with those with a normal diameter colon.

HRCM showed striking differences between those with enlarged colons vs those without. As figure 4 shows patients with an enlarged colon at baseline failed to show the normal increase in PW centred around three cps after a meal seen with the remaining subjects.

**Figure 5:**
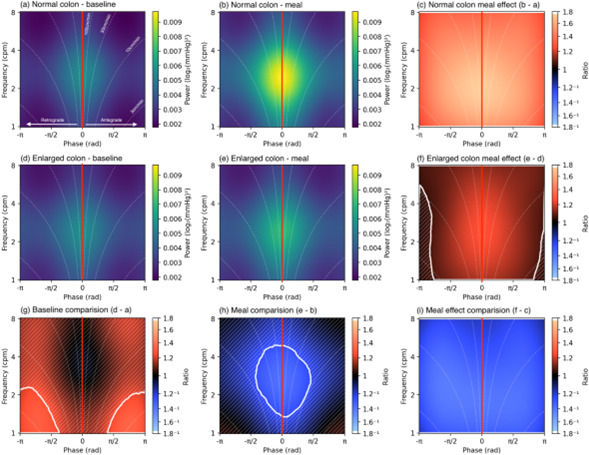
Two-dimensional (2D) analysis of propagating pressure waves (PPW) in the sigmoid colon at frequencies between 1 and 8 cpm. In each panel, the vertical line at 0 on the x-axis indicates synchronous (non-propagating) activity. Retrograde propagation is to the left of the midline and antegrade to the right. The curved dotted lines indicate the speed of propagation, from 3 cm/min to 100 cm/min. (**a, b, d, e**) The green pixels represent the increasing power of propagated activity. The first column represents baseline data, the second column meal data. Patients with a normal diameter colon are shown in the top row, patients with an enlarged colon in the second row. The bottom row compares PPW power across the frequency range between the normal and enlarged colon during baseline (**g**) and meal (**h**) periods. (**g**) The orange area demarcated by the solid white line indicates a significant increase in antegrade and retrograde PPW at <3 cpm in the enlarged compared with normal volume colons. (**h**) The blue area demarcated by the solid white line indicates a significant decrease in antegrade and retrograde PPW between 2 and 4 cpm in patients with enlarged compared with normal volume.

Examining the PW in more detail using the 2D analysis which analyses the PPWs, both retrograde and antegrade, similarly shows that the enlarged colons fail to show a meal-related increase on both retrograde and antegrade propagated contractions (figure 5).

### Manometry versus MRI

There were no significant correlations between the MRI measures of CVs, AC MMI, tagging index or WGTT and the percentage time occupied by CMPs (online supplemental G3 table S5). Classifying participants as hypomotile by MRI (<10th centile of MMI) or manometry (<10th centile of CMPs) showed little agreement and only one subject was hypomotile by both criteria (online supplemental G4 table S6).

## Results Part 2: randomised, placebo-controlled trial comparing bisacodyl and hyoscine

### Clinical characteristics

Two patients only completed one arm of the cross-over (one missed bisacodyl and the other hyoscine) leaving 70 sets of paired results. Demographics and bowel habits are shown in online supplemental H1 table S7 and were balanced in both treatment sequences (online supplemental H2 table S8).

## Results

Overall, hyoscine was better tolerated than bisacodyl without difference between the two groups. Only 1 FC and 1 IBS-C taking hyoscine reduced dose due to side effects and no patients stopped early while 26 patients required dose adjustment with bisacodyl (10 FC, 16 IBS-C) with 5 stopping early (2 FC, 3 IBS-C).

The primary endpoint, namely the difference in average worst daily pain scores on bisacodyl versus hyoscine, had a median value (range) of 0.3 (−0.2, 0.8) in IBS-C and 0.7 (−1.2, 1.4) in FC, a difference which was not significant, p=0.2. The correlations between the difference in average worst daily pain scores on bisacodyl and hyoscine and AC MMI and tagging index at 120 min were not statistically significant (Pearson r=−0.16 and 0.13, p=0.22 and p=0.32 for MMI and tagging index, respectively).

Bisacodyl was more effective in both IBS-C and FC in increasing the median number of CSBMs compared with hyoscine. Stools were significantly softer on bisacodyl and the number of days with hard or no stool was significantly less in both groups (table 4). Only 8 participants (5 FC, 3 IBS-C) on bisacodyl required rescue therapy (prucalopride, senna or picosulphate according to patient preference) vs 18 on hyoscine (9 FC, 9 IBS-C). However, both average worse daily pain and mPAC-SYM scores were higher for all patients when taking the bisacodyl (table 4) and for both FC and IBS-C (online supplemental H3table S9).

**Table 4:** Clinical endpoints of RCT of bisacodyl versus hyoscine (all patients: n=70 paired) median (IQR)

	Bisacodyl	Hyoscine	P value
Pain			
Average worst daily pain (range 1–5)	2.3 (1.8–3.1)	1.6 (1.3 to 2.3)	<0.001
mPAC-SYM (abdominal pain, discomfort and cramps) after intervention(range 0–4)	2 (1.3–3)	1 (0.3 to 2)	<0.001
Stool frequency and consistency (over 10-day period)			
CSBM	4.0 (0.0–9.0)	0.0 (0.0 to 2.0)	<0.001
Average BSFS over the 10 days (excluding BMs following rescue, BSFS 1–7)	5.3 (4.7–6.0)	2.4 (1.3 to 3.8)	<0.001
Days with hard (BSFS one or 2) or no stool, or needing rescue	2 (1–5)	5.6 (3–7)	<0.001

BSFSBristol Stool Form ScaleCSBMcomplete spontaneous bowel movementsmPAC-SYMmodified Patient Assessment of Constipation-SymptomsRCTrandomised controlled trial

Considering IBS-C and FC separately, both groups responded similarly to bisacodyl with more CSBM and softer stools compared with both baseline and hyoscine which in contrast produced no significant changes in any of our endpoints (online supplemental table S9). However IBS-C participants reported significantly higher average worse pain compared with FC on bisacodyl, values being 2.7 (2.1–3.3) vs 2 (1.6–2.5) but pain on hyoscine was not significantly different being (1.7 (1.4–2.7) vs 1.5 (1–2), for IBS-C and FC, respectively (mixed-effects ANOVA, effect of treatment F=38.9, p<0.0001, effect of group F=9.4, p=0.003, interaction term not significant).

### Impact of MRI and manometry outcomes on response to treatment

Non-responders to bisacodyl (those that failed to increase CSBM by >1) did tend to have larger baseline volumes (893±286, n=33 vs 774±251, n=36, p<0.07). Thus only 33% of patients with enlarged colons were bisacodyl responders v 59% of those with normal colon volume but again this just failed to reach significance, Fisher’s exact test p=0.09.

### Impact of enlarged colon on response to treatment

Those with an enlarged colon had significantly less increase in pain as assessed by mPAC-SYM on bisacodyl. They tended to have fewer CSBMs but this was not significant (table 5). There was no difference in response to hyoscine (online supplemental H4 table S10).

**Table 5:** Effect of enlarged colon on response to bisacodyl

	n	Basal mPAC-SYM	Change in mPAC-SYM	Weekly CSBM	Change in BM
Enlarged colon	22	1.2(0.8)	0.6(1.0)	1.4(0.0–3.7)	0.5(0.0–2.8)
Normal-sized colon	49	1.1(0.8)	1.0(0.9)	2.3(0.0–5.3)	2.3(0.0–5.3)
P for difference		0.6*	0.05*	0.06‡	0.12‡

CSBMcomplete spontaneous bowel movementmPAC-SYMmodified Patient Assessment of Constipation-Symptoms

## Discussion

MRI provides a novel approach to assessing colonic function, the utility of which this study attempted to determine. Despite disproving some of our original hypotheses we were able to show that constipation is associated with an enlarged colon and that those with colon size exceeding the 90th centile of HVs (33% of our constipated cohort) did show a delay in defaecation after macrogol administration and significantly impaired motor response to feeding. They also had significantly less pain and a tendency to less CSBMs with bisacodyl. The significance of an enlarged colon complements studies in constipated paediatric patients showing sigmoid dilatation in a proportion of sufferers in whom underlying organic pathology has been ruled out,^25^ and also extends a growing body of literature demonstrating that rectal hyposensitivity (present in 25% of constipated adults)^26^ is secondary to an enlarged or hypercompliant rectum in the majority.^27^

The macrogol challenge, which approximately doubles CVs, is designed to be a substantial reproducible stimulus to proximal colonic motility,^12^ something our current study confirms. Although it enables us to non-invasively assess the motility of both the ascending and DC, our study shows that this did not correlate with the response to a meal using high-resolution manometry of the distal colon. However, HRCM is a difficult technique and there are considerable obstacles to using it widely in clinical practice including the availability and expense of the equipment and the patients’ dislike of invasive procedures. Although MRI after macrogol cannot produce the same details as HRCM its convenience and high patient acceptability may lead to it being more widely used in the future.

This large study recruiting from both primary and secondary care in multiple sites found CVs and WGTT were highly variable and did not differ between FC and IBS-C, though both were significantly greater than HVs. Thus, we did not confirm our earlier smaller study suggesting that IBS-C had smaller colons possibly because this previous study recruited extremes from tertiary care less representative of general clinical practice.^11^ IBS-C did, however, have a lower tagging index after macrogol suggesting their motor response was less efficient at moving colonic contents. However, the main difference was pain (IBS-C>FC) both at baseline and 60 min after macrogol as well as during bisacodyl and hyoscine treatment.

HRCM is more demanding for both patient and investigator and less widely available but can provide very detailed information on colonic contractile activity. This is one of the largest such studies and our data showed that while a meal resulted in a significant increase in PW in all three groups, the coordination of these PW into PPW was significantly reduced in IBS-C patients, compared with both HV and FC. Uncoordinated contractions particularly during the postprandial period when there is an increase in contractile activity could cause pain in IBS patients, but this requires further study.

We had hypothesised that the difference in pain score on a stimulant (bisacodyl) versus a smooth muscle relaxant (hyoscine) would be greater in those with hypermotility, but in the event, it did not correlate with either MMI, tagging index, WGTT nor HRCM. We did, however, show that although bisacodyl is an effective laxative, it does increase pain in IBS patients more than hyoscine, which in contrast did not alter any of the recorded symptoms and required rescue laxatives for most of our patients.

Limitations of this study include the fact that for both expense and patient comfort reasons using MRI one can only record motility from the colon for short periods using the macrogol challenge. However, from HRCM studies, we know that colonic motility is erratic and needs prolonged recording to get reliable results. Furthermore, the mechanism of response to the distension induced by macrogol is quite different from the more physiological response following the meal we used in the HRCM study, which probably accounts for the lack of correlation between the two measures. Another concern relates to the image registration of successive cine images required to overcome artefact generated by the movement of the diaphragm and abdominal contents in a free-breathing subject. While this works well with relatively shallow breathing, large deviations of the diaphragm can cause changes to the colon wall that are not associated with wall contractions, leading to an artificial increase in the MMI. The tagging technique does overcome this limitation as it is a breath-hold scan and may be a more reliable measure though it does assess the movement of chyme rather than wall movement per se and also over a much shorter duration. This insight should be further investigated in large clinical cohorts to test its utility and ability to predict response to treatments.

Previous assessments of CV in vivo have either used the volume required to fill a colon during barium enema or used ionising radiation (X-ray/CT scanning). MRI provides a much more acceptable way of assessing volume in the undisturbed colon. The ability to assess specific regional volumes may prove an advantage when dealing with the rare but difficult to manage patients with severe constipation and underlying megarectum and/or megacolon since it may guide the choice of surgical or medical therapies.^28^ The underlying pathophysiology of an enlarged colon remains to be determined but this can be assessed using normal MRI scanners available in many hospitals. Further investigation of the causes of constipation including the association between an enlarged colon and manometry will require larger numbers but could easily include CV assessed using MRI. The development of MRI-compatible fibreoptic manometry tubes^29^ will allow the simultaneous imaging and pressure measurement of the range of manometry patterns including HAPCs. While waiting for spontaneous or meal-induced HAPCS is not feasible with MRI owing to their low frequency (approximately 4–5 per day^30^), expense and patient discomfort related to prolonged scanning, an agent like bisacodyl, which produces a rapid response,^31^ will make such studies possible. These will allow a non-invasive assessment of the impact of HAPCs on colonic tone, motility and contents and also identify MRI patterns characteristic of patients who fail to respond to bisacodyl. Future studies could also include novel prokinetic agents to allow better evaluation of their mode of action.

## supplementary material

10.1136/gutjnl-2024-332755 online supplemental file 1

10.1136/gutjnl-2024-332755 online supplemental file 2

10.1136/gutjnl-2024-332755 online supplemental file 3

10.1136/gutjnl-2024-332755 online supplemental file 4

10.1136/gutjnl-2024-332755 online supplemental file 5

10.1136/gutjnl-2024-332755 online supplemental file 6

10.1136/gutjnl-2024-332755 online supplemental file 7

10.1136/gutjnl-2024-332755 online supplemental file 8

10.1136/gutjnl-2024-332755 online supplemental file 9

10.1136/gutjnl-2024-332755 online supplemental file 10

10.1136/gutjnl-2024-332755 online supplemental file 11

10.1136/gutjnl-2024-332755 online supplemental file 12

## Data Availability

Data are available on reasonable request.
