# Supplementary figures and images for: Combined MRI, high-resolution manometry and a randomised trial of bisacodyl versus hyoscine show the significance of an enlarged colon in constipation: the RECLAIM study
Source: Gut. 2024 Oct 22;74(1):e332755. doi: 10.1136/gutjnl-2024-332755 (PMC11671975; doi:10.1136/gutjnl-2024-332755)

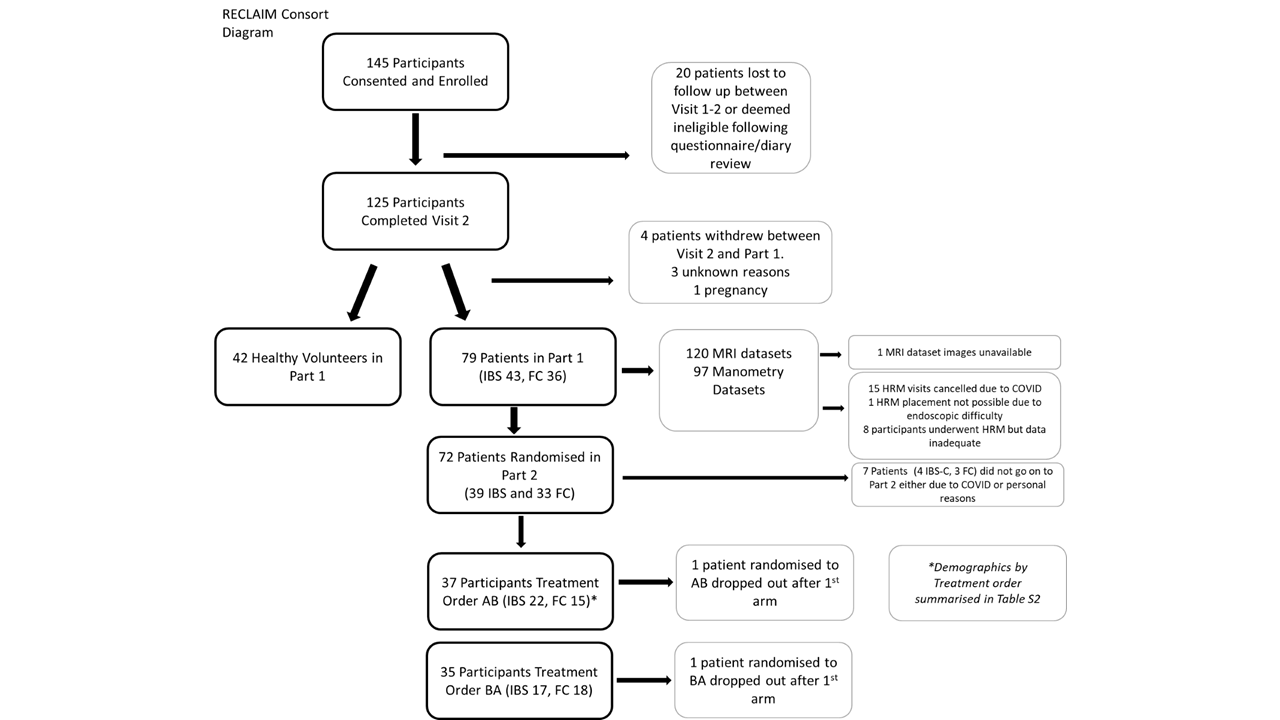

Supplement: online supplemental file 1 [file gutjnl-74-1-s001.tif]

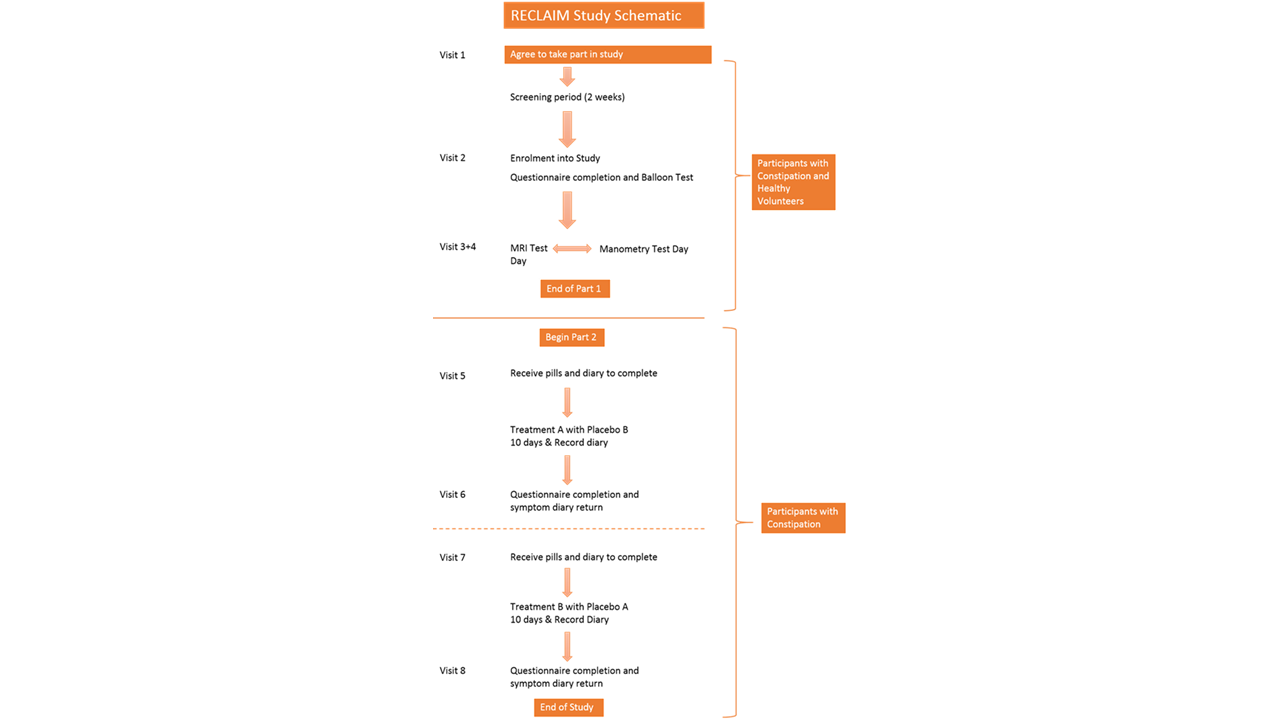

Supplement: online supplemental file 2 [file gutjnl-74-1-s002.tif]

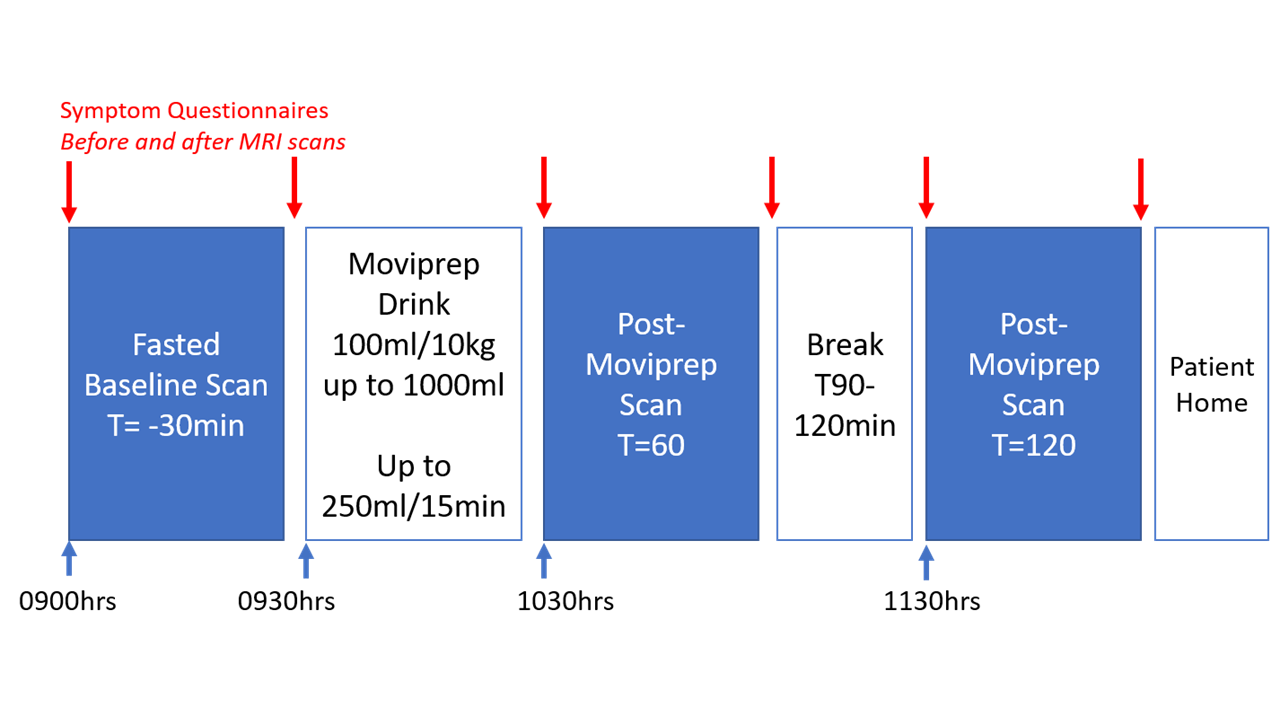

Supplement: online supplemental file 3 [file gutjnl-74-1-s003.tif]

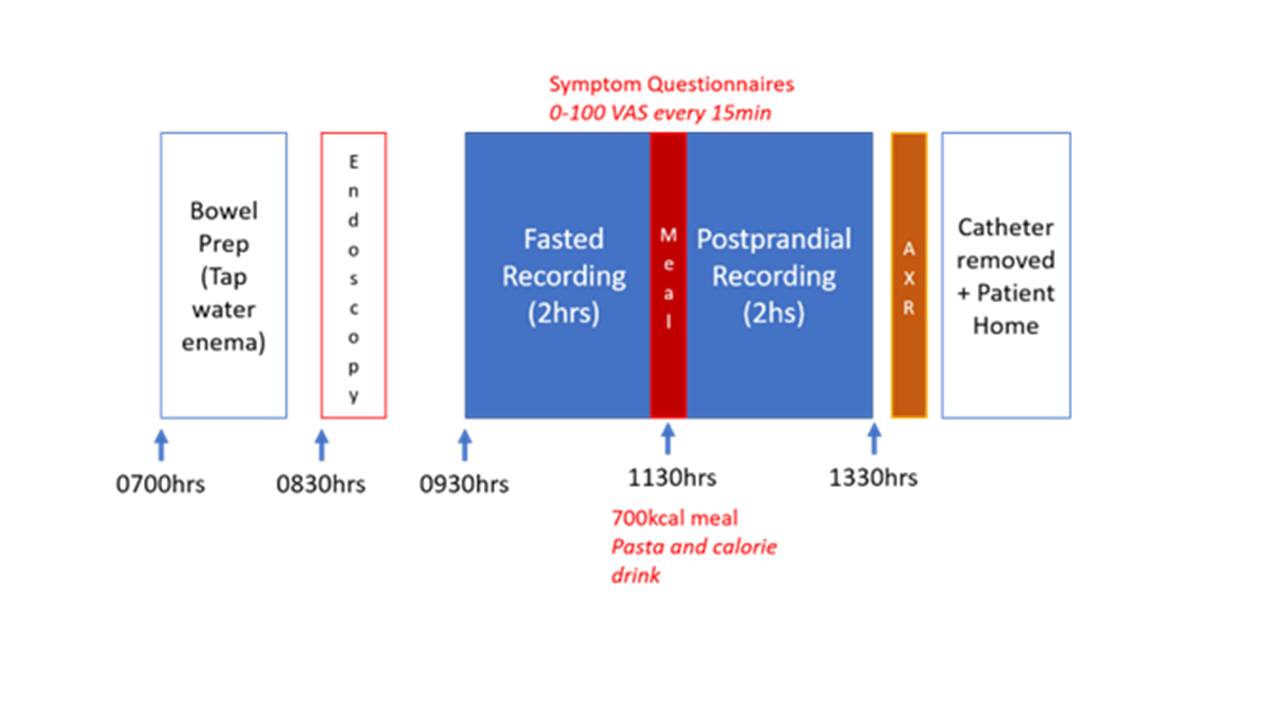

Supplement: online supplemental file 4 [file gutjnl-74-1-s004.tif]

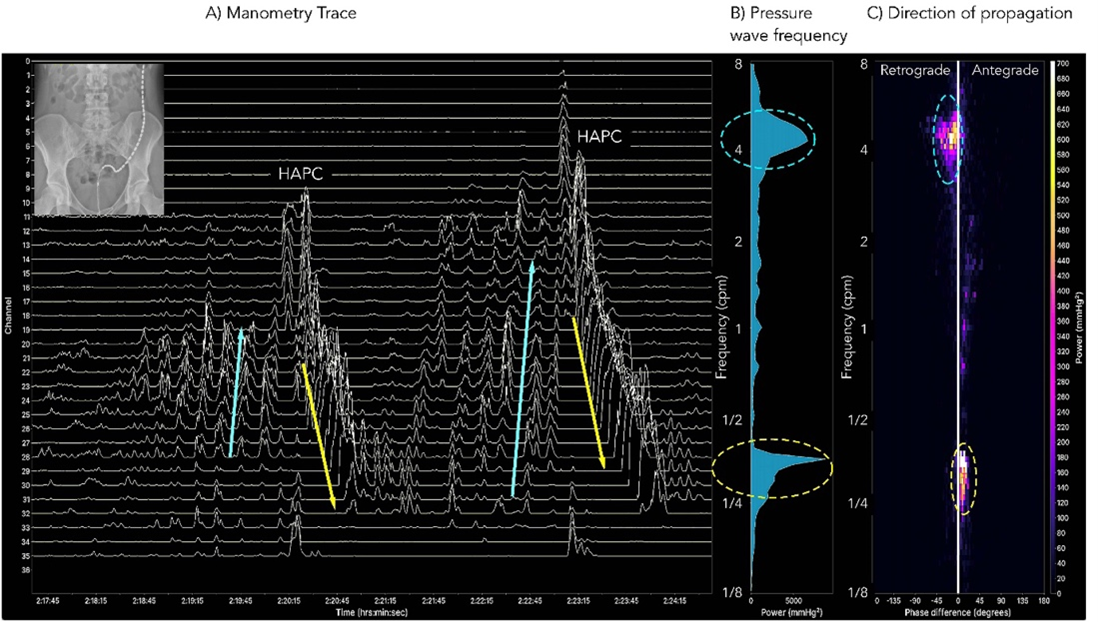

Supplement: online supplemental file 5 [file gutjnl-74-1-s005.tif]

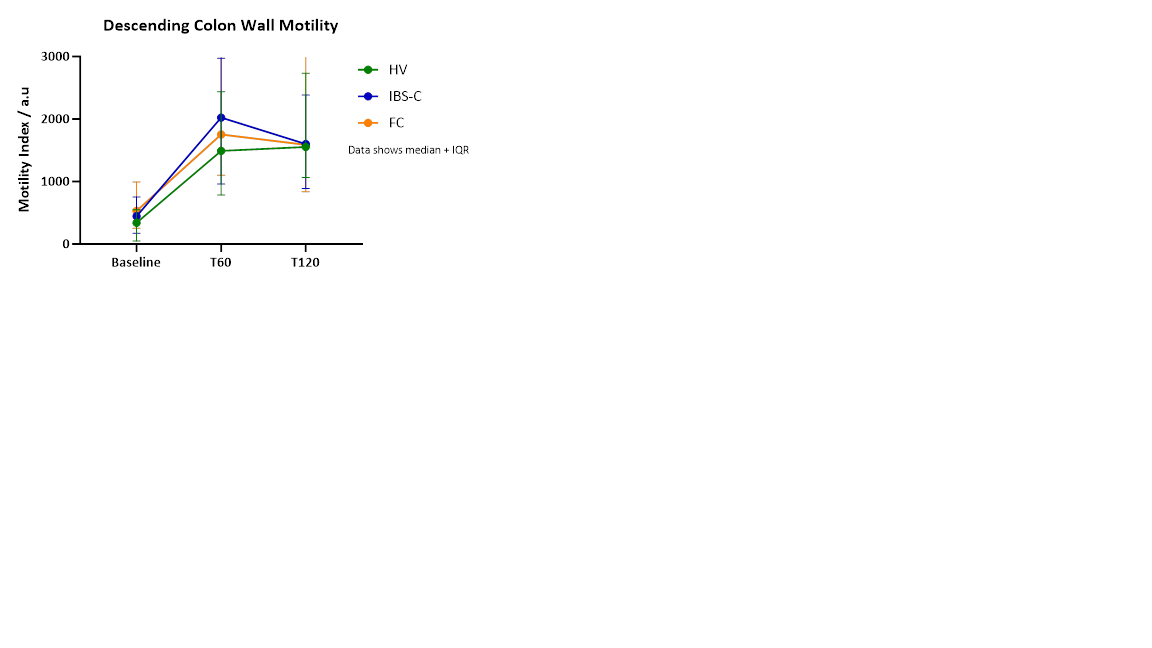

Supplement: online supplemental file 6 [file gutjnl-74-1-s006.tif]

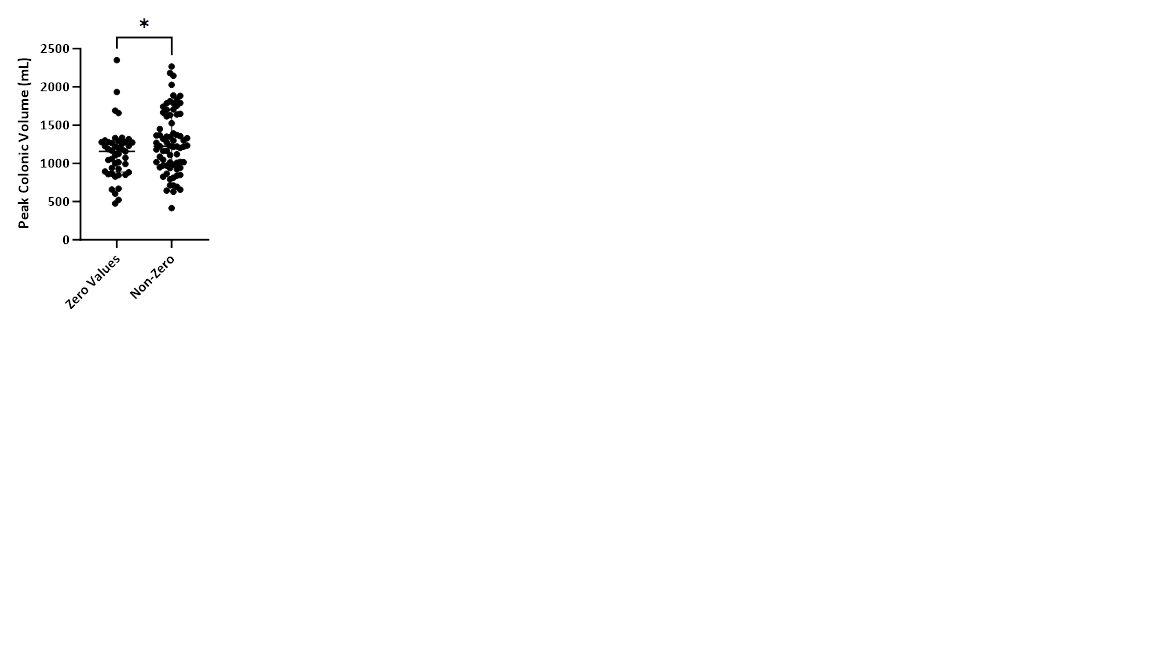

Supplement: online supplemental file 7 [file gutjnl-74-1-s007.tif]

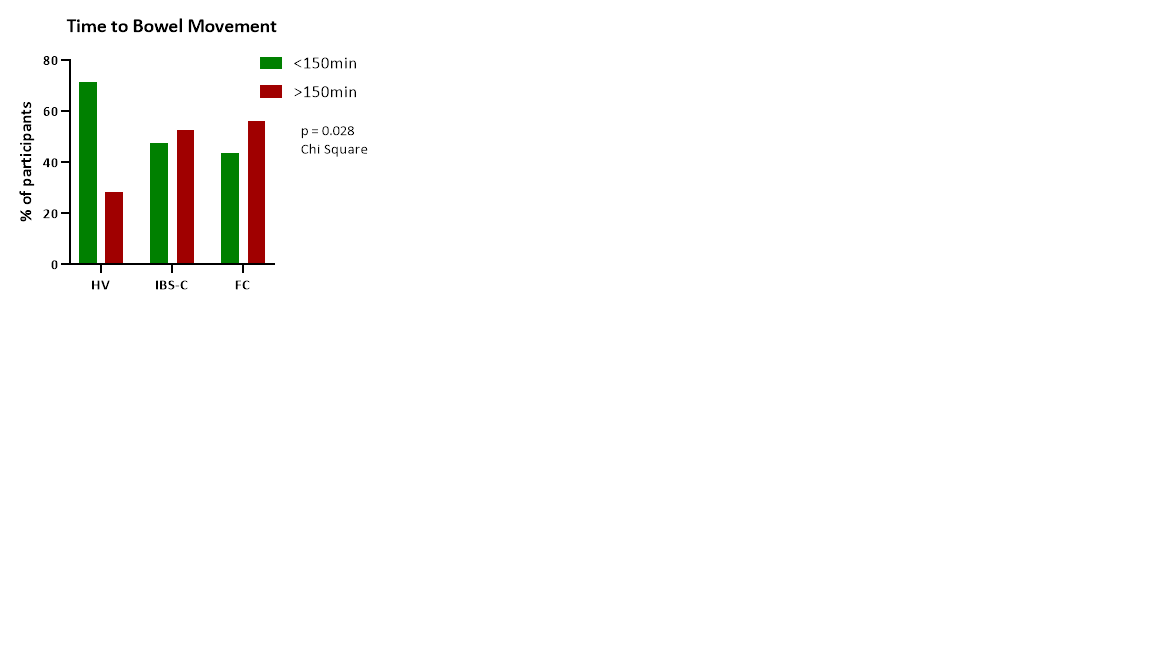

Supplement: online supplemental file 8 [file gutjnl-74-1-s008.tif]

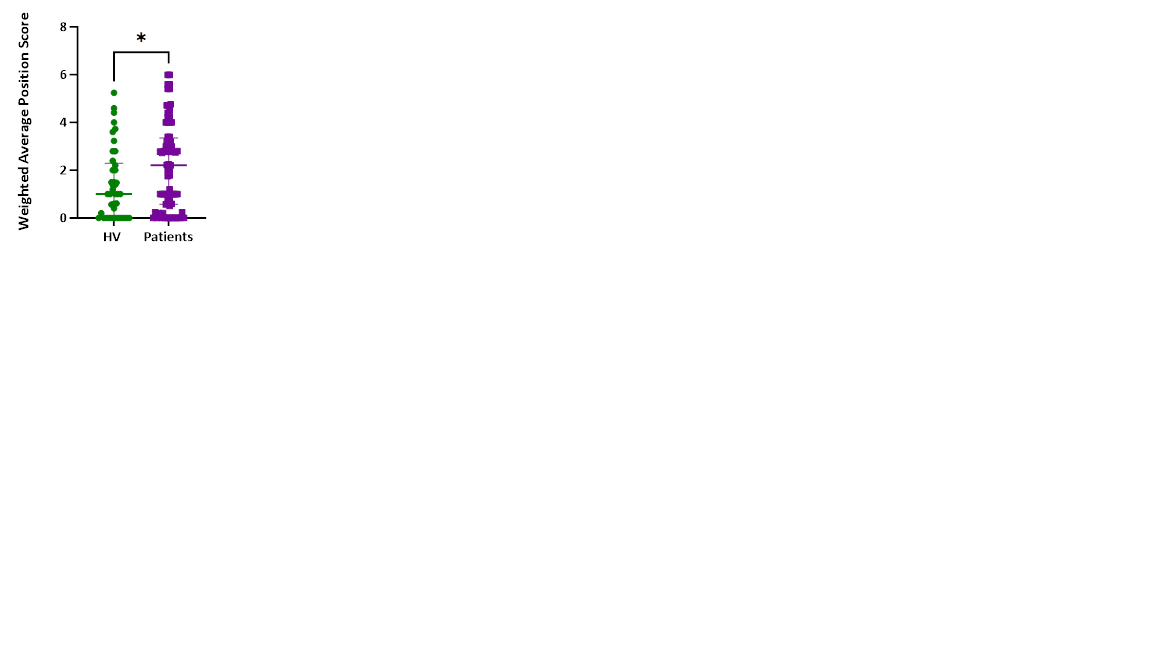

Supplement: online supplemental file 9 [file gutjnl-74-1-s009.tif]

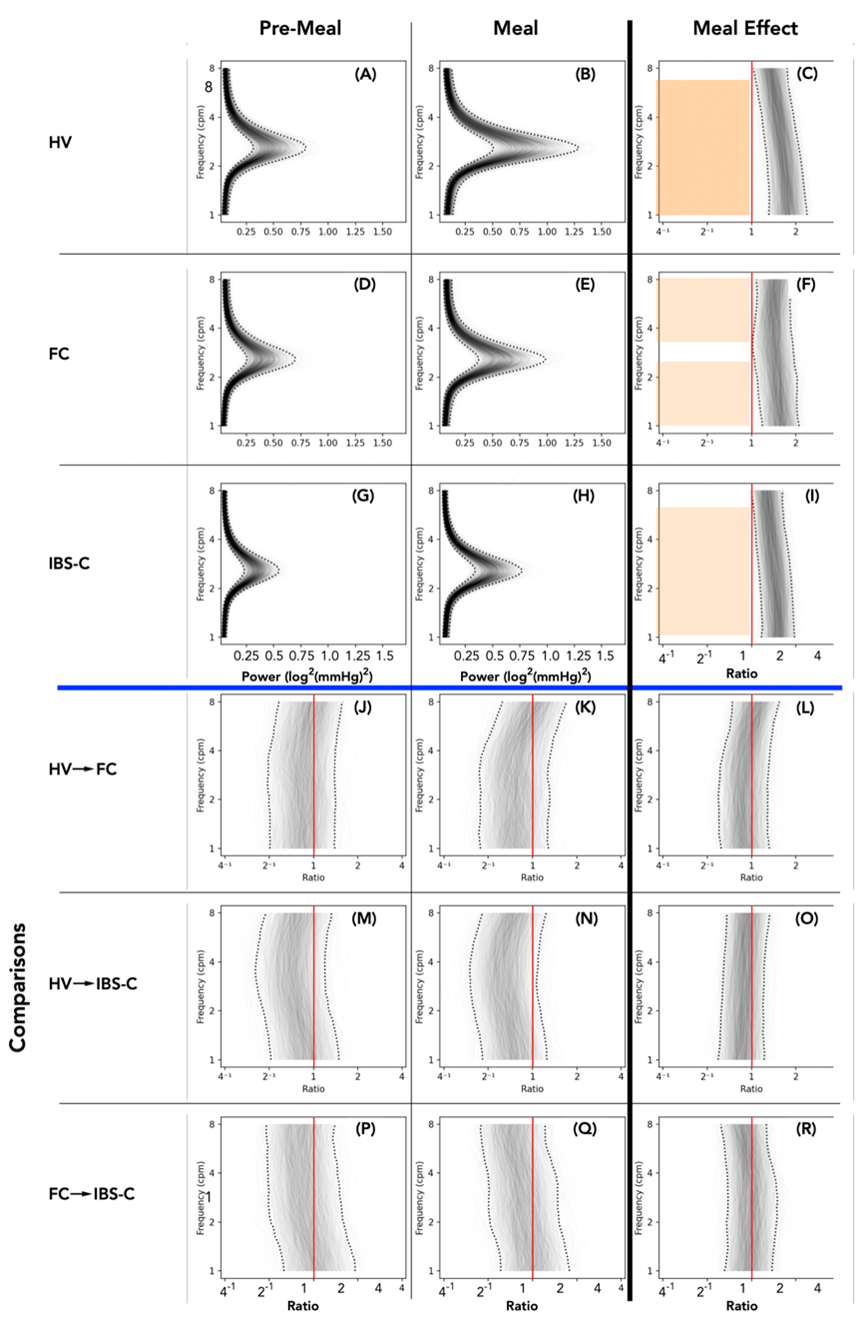

Supplement: online supplemental file 10 [file gutjnl-74-1-s010.tif]

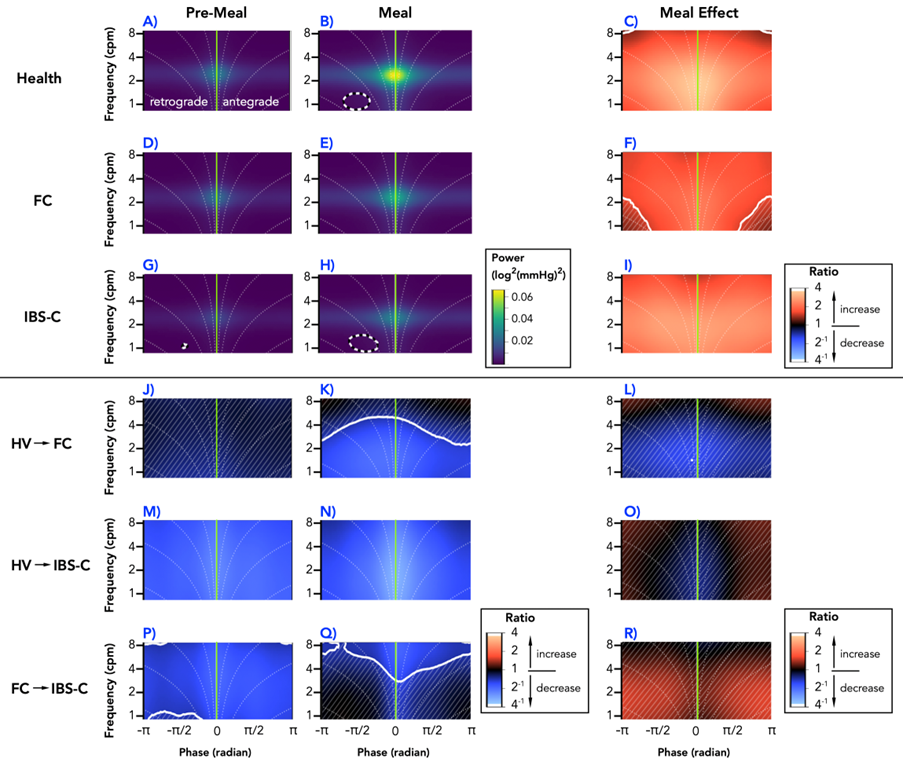

Supplement: online supplemental file 11 [file gutjnl-74-1-s011.tif]
