# Supplementary material for: Combined MRI, high-resolution manometry and a randomised trial of bisacodyl versus hyoscine show the significance of an enlarged colon in constipation: the RECLAIM study
Source: Gut. 2024 Oct 22;74(1):e332755. doi: 10.1136/gutjnl-2024-332755 (PMC11671975; doi:10.1136/gutjnl-2024-332755)
Supplement: online supplemental file 12 [file gutjnl-74-1-s012.docx]

Supplementary data

# A Methods

# A. Recruitment

Participants with constipation and healthy volunteers were recruited at 2 sites in the UK (Nottingham and London) from both primary and secondary care, and also through social media. After consent we confirmed potential eligibility as either a HV or a patient with constipation using the Cleveland Clinic constipation Score (HV cut off was ≤5). Those whose diary confirmed they had < 3 complete spontaneous bowel movements (CSBM) per week on average were enrolled as patients. HVs were required to have >3 CSBMs per week on average. Constipated patients were classified into either FC or IBS-C based on their responses to the ROME IV criteria questionnaire.

## **A.1 Study Eligibility Criteria**

**Inclusion criteria**

1. Aged ≥ 16 years

2. Capacity to give informed consent for participation

3. Ability to understand written and spoken English

4. For Constipation Group: Symptoms of constipation meeting Rome IV criteria for functional constipation or constipation-predominant irritable bowel syndrome

5. For Control Group: No symptoms of constipation. This will be defined as a score of 5 or less on the Cleveland Clinic Score

**Exclusion criteria**

1. Participation in any clinical trials in the past 3 months

2. Inability to understand written and spoken English

3. Pregnancy, assessed by a urinary pregnancy test, or current breastfeeding

4. History of significant adverse reaction or hypersensitivity, or known contra-indication to any of the medicinal products or equipment used in the study

5. History declared by the candidate of certain pre-existing gastrointestinal disorders, including:

i. inflammatory bowel disease

ii. coeliac disease

iii. cancer of the gastrointestinal tract

6. Any reported history of gastrointestinal resection (excluding appendicectomy or cholecystectomy)

7. Presence of an intestinal stoma

8. Causes of secondary constipation disorders (e.g., systemic sclerosis / Parkinson’s disease)

9. Inability to cease use of medicines that cause constipation or alter colonic contractility (e.g., opioids, smooth muscle relaxants)

10. Antibiotic use in the last 3 months

11. Comorbidity that would prevent safe adherence to the protocol (e.g., inability to lie flat, kidney disease contraindicating use of Moveprep)

12. Judgement by the PI that the candidate who will be unable to comply with the full study protocol (e.g., diabetes, severe COPD)

13. Contraindication to MRI or colonic manometry

• Examples for MRI include claustrophobia, metallic implants, pacemakers, history of metallic foreign body in eye(s) and penetrating eye injury

• Examples for manometry include diagnosis of previous complications of diverticular disease or previous endoscopic complications

14. Contraindication to Medicines to be used in study

• Examples include prostatism or glaucoma

15. Clinical evidence of significant pelvic organ prolapse syndromes

16. Inadequate screening diary following review

i. Control Group: A screening diary that records <6 complete spontaneous bowel motions in the fortnight.

ii. Constipation Group: A screening diary that records >6 complete spontaneous bowel motions in the fortnight

## A 2 Participant flow

As reported in the Consort Diagram in Figure S1 below, a total of 145 participants were consented and enrolled across both sites, of whom 125 eligible participants completed Visit 2 (HV 44, IBS 43, FC 38). Recruitment started August 1^st^ 2017 and the last patient completed December 10 2020

121 participants then proceeded to complete at least one visit of Part 1 of the study (HV 42, IBS 43, FC 36). 72 patients with constipation (39 IBS and 33 FC) then went on to participate in Part 2.

## A.3 Figure S1: Consort Diagram

  
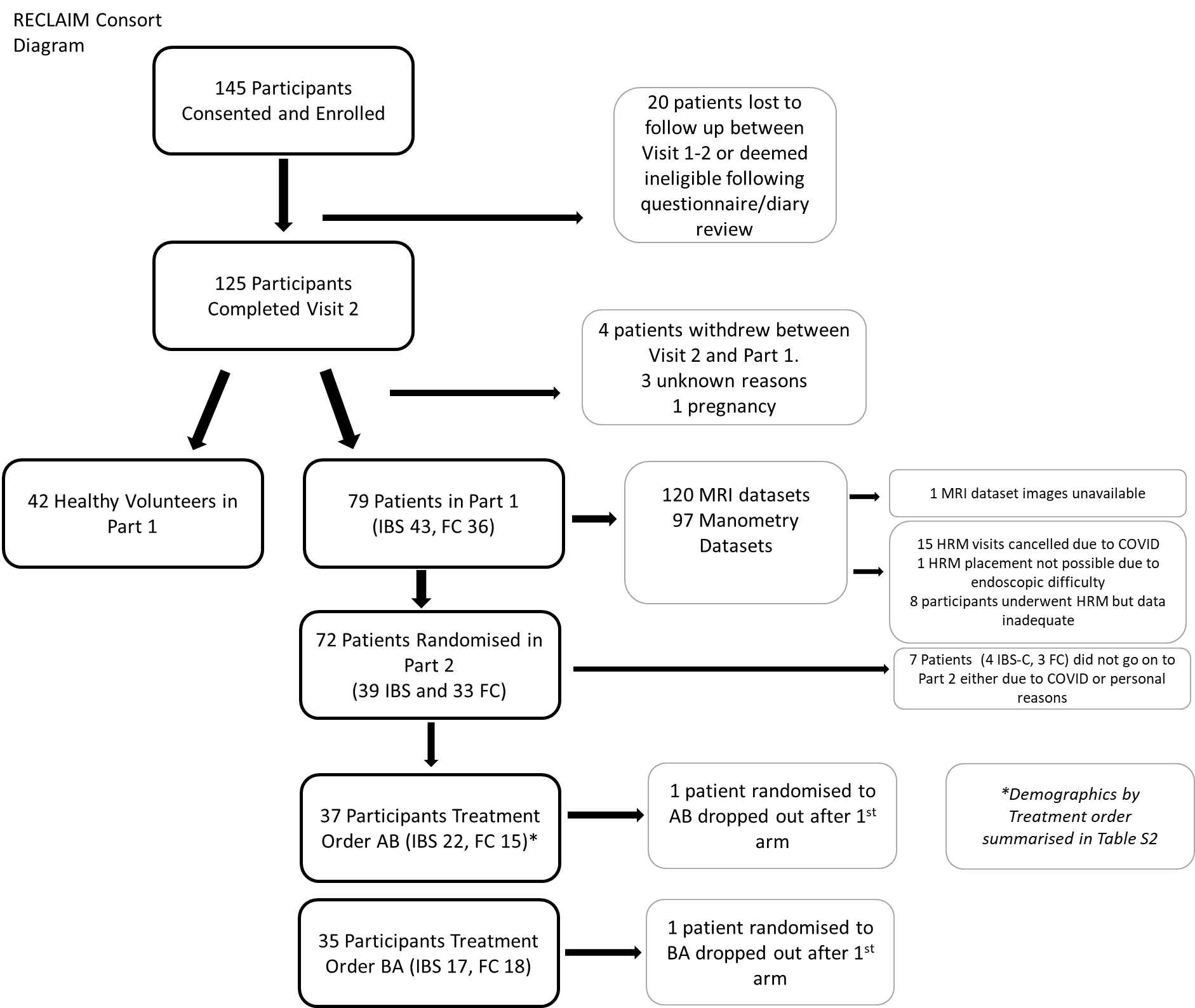


# B. Study Visit Protocols

The study took place over 8 visits. The first visit was to check eligibility and consent before the screening period commenced.

All participants completed a daily study diary documenting whether they had attempted to open their bowels, whether they had passed stool, the number of bowel movements (BMs) in the day, the stool form score for each BM and whether the bowel movement was a complete spontaneous bowel movement (CSBM), defined as “a bowel motion with the feeling of complete evacuation, without using your laxative/rescue therapy”. They also recorded if they had taken any rescue medication.

At the second visit the results of the diary were reviewed and, if eligible, the participant completed the HADS and PAC-SYM questionnaire and underwent the balloon expulsion test.

## B1 Balloon expulsion test

This was completed using a 50mL water filled balloon, and the patient allowed up to 2 mins to attempt in the seated position with adequate privacy.

Visits 3 and 4 were the MRI and manometry visits, details outlined below. These were performed in a random order.

Visits 5-8 involved the crossover trial, with an initial visit to dispense the blinded tablets and questionnaire, a 10-day treatment period, followed by a minimum 7-day wash-out period before commencing the second set of tablets. The study visits are summarised in Figure S2.

## B.2 Figure S2: Study Schematic
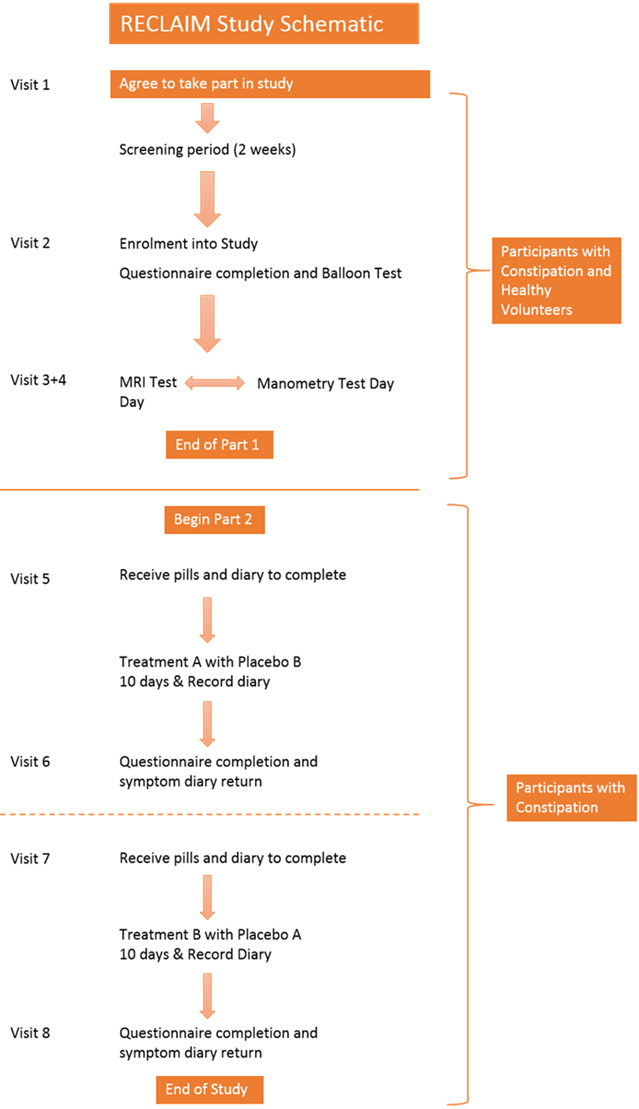


## B.3 Table S1: Baseline Data on Bowel Habit and Balloon Expulsion Test

|  | HV (n=44) | IBS-C (n=43) | FC (n=38) | |  | |
| --- | --- | --- | --- | --- | --- | --- |
| Screening Stool Diary (14-day diary) | | | | p | |  |
| Total BM Attempts | 17±7 | 16±13 | 18±18 | | 0.743 | |
| Total BM Inc those after rescue medication | 17±7 | 13±11 | 11±9* | | 0.17 | |
| Number of SBM | 17±7 | 12±12 | 10±11* | | 0.05 | |
| Number of CSBM | 15±7 | 1±2* | 2±3* | | <0.01 | |
| Days with hard (BSFS 1 or2) or no stool | 2±3 | 12±3* | 12±3* | | <0.01 | |
| Average stool consistency ‡ | 4±1 | 2±1* | 2±4* | | <0.01 | |
| Balloon expulsion test result | | | |  | |  |
| BET (% Pass) | 89% | 84% | 75% | | 0.27 | |

# C MRI Protocol

The participants were provided with 5 plastic MRI visible marker pills to swallow 24 hours before attending the local test site at 9am on the morning of the test. They did not eat or drink anything from the night before, apart from sips of water for essential medicines. The patient cohort were also asked to refrain from laxatives for 48hrs prior to the visit. Participants completed an eligibility questionnaire to confirm the above and that there were no new contraindications to MRI before undergoing the first series of MRI scans (outlined below) while fasted, lasting approximately 30 minutes.

30 minutes after the start of the scan participants started drinking a poly-ethylene glycol and electrolyte solution (Moveprep^R^, Norgine Pharmaceuticals Ltd, Harefield, UK). They drank 10ml/kg body weight, rounded to the nearest 100ml within the 500ml-1000ml range, ¼ of the total to be drunk every 15 minutes. Moveprep^R^ 1 litre contains 100 g macrogol with 182 mmol Na+, 52.8 mmol SO4, 59.8 mmol Cl-, 14.2 mmol K+ and 56.5 mmol ascorbate.

Immediately after finishing the Moveprep^R^, participants had a further set of MRI scans (T=60). A further scan then taken an hour later (T=120). The time to first bowel movement after drinking the Moveprep^R^, was also recorded wherever possible. If participants did not open their bowels during the study session, they were asked to note this and inform us, if they did not this was recorded as >150mins.

Imaging was carried out on a 3.0T Ingenia wide-bore scanner (Philips, Best, The Netherlands) with a parallel imaging SENSE abdominal body receiver coil.

### C1 MRI analysis

The principal MRI techniques and analyses were as previously described:

1. MRI colonic volumes. A 3D coronal dual echo fast field echo sequence with mDIXON reconstruction. (1)
2. MRI wall motility measurement. Cine bTFE data were acquired over 10 minutes (2 ,3).
3. MRI content mixing measurement – “tagging index”. A single slice cine bTFE with tag lines 12 mm apart was acquired over a 20 s breath-hold positioned oblique-sagittally through the AC. This acquisition was also repeated oblique-coronally. Variation in the pixel intensity was assessed from the average coefficient of variation (%COV) for ascending colon which is the “tagging index”. (4)
4. completed tagged scans of the ascending colon.
5. MRI transit measurement. Whole Gut Transit Time (WGTT) was assessed from the Weighted Average Position Score (WAPS) of MRI transit markers 24 hours after ingestion, as we have previously validated, where a higher score equates to a longer transit time.(5) This was assessed at the baseline scan before ingestion of the Moviprep^R^.

Additional sequences were taken in order to position the scan.

### C 2 Figure S3: Schedule for MRI study day


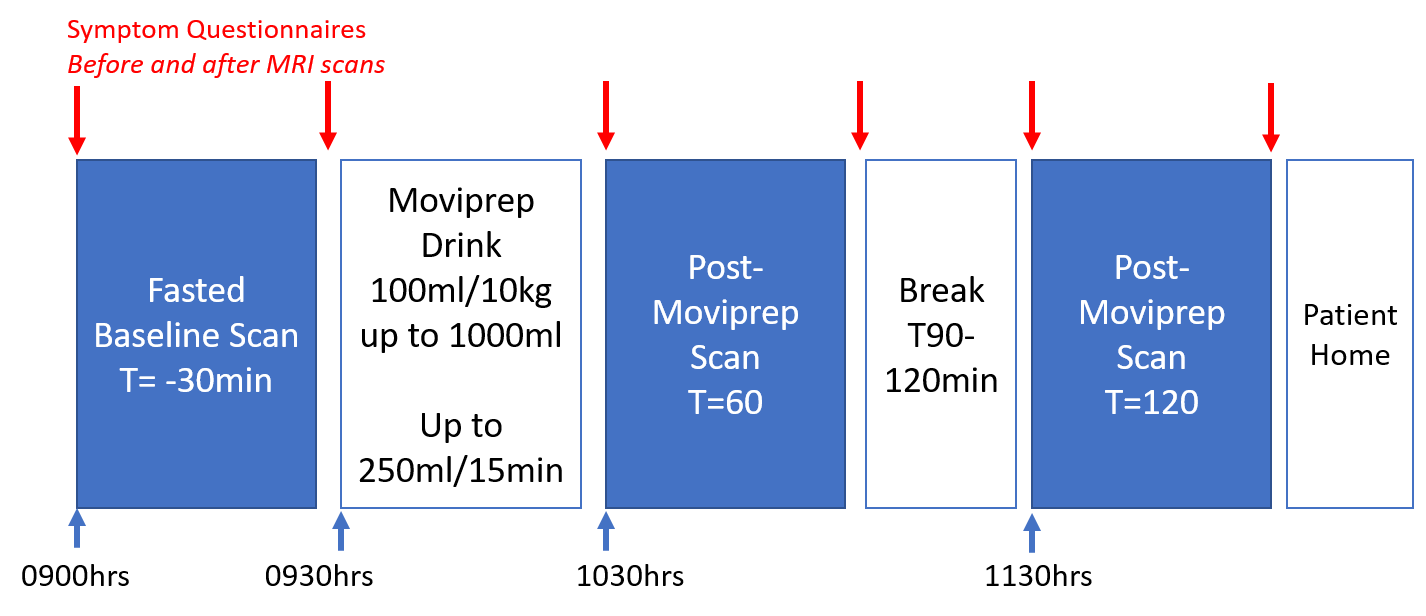


### C 3 Manometry Protocol

On the morning of the test the participant attended the local test site, fasted from the night before, apart from sips of water for essential medicines. Prior to the endoscopy procedure a tap water enema was administered to cleanse the left side of the colon. Once the study team were satisfied that bowel preparation had been satisfactorily achieved, the participant was transferred to an endoscopy procedure room to undergo colonoscopy. The colonoscope was then passed and the flexible manometry catheter (~4 mm in diameter with 36 recording sites spaced 1 cm apart) was inserted alongside it. Once in place, about 35 cm into the bowel, a thread attached to the tip of the catheter was secured to the lining of the bowel with a fine metal clip, deployed through the colonoscope. Once the colonoscope was removed, the catheter was taped in place at the anus using an adhesive dressing.

After approximately 30 mins (to allow for a recovery period following colonoscopy), and with the participant in a semi-reclining position on a hospital bed, the catheter was connected to the recording equipment and monitoring commenced. Following a 2 hour ‘rest’ (baseline) period, the participant was supplied with a standard 700 Kcal meal and asked to consume this within 10 minutes. The meal consisted of a 200 mL of Ensure TwoCal (Abbotts Nutrition) nutrient drink (400Kcal) and a pasta meal (300Kcal).

The recording was then continued for a further 2 hours. At the end of the recording period the participant was transferred by wheelchair to have an abdominal X-ray. This confirmed the position of the catheter. They then returned to the investigation room and the catheter was removed by gentle traction.

For the high-resolution manometry, the catheter and software system used was purchased from Medical Measurement Systems. This consists of a catheter which was inserted endoscopically and a software system for collection of data. The catheter used was the “Unisensor UniTip High Resolution Catheter” which is CE-marked for non-surgical invasive use in the area of Gastroenterology. As this manometry system is CE marked and being used within its intended purposes we did not require a letter of no objection from the MHRA for usage.

### C4 Figure S4: Schedule for manometry study day


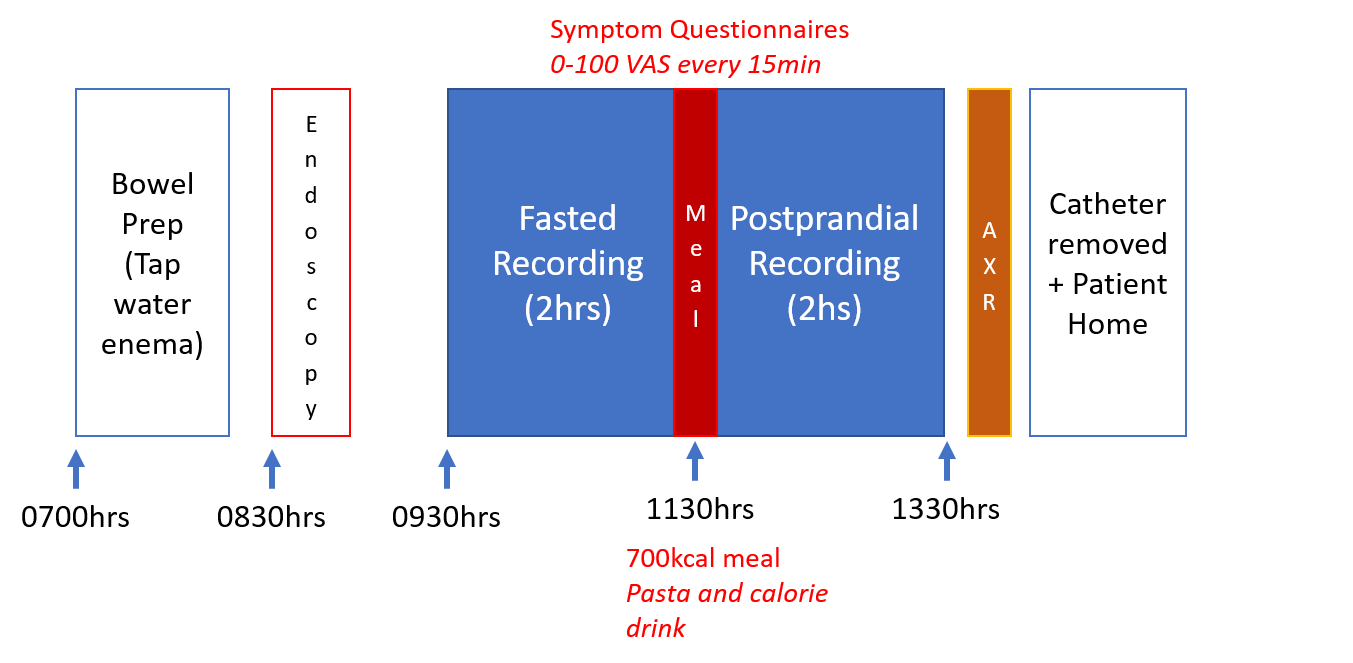


## D. Clinical Trial Protocol

The interventions used were Buscopan 20mg taken orally three times daily for 10 days and bisacodyl 10mg taken orally once daily for 10 days. We provided 2 sets of capsules: A and B. One of the capsules contained either Buscopan or Bisacodyl and the other a placebo. Whichever capsule was the placebo in the first 10 days trial was replaced by the active drug in the second part. The order in which they were taken was randomised according to a code generated by the pharmacy team at Nottingham University Hospitals NHS Trust (lead pharmacy site) so neither participant nor the research workers knew which active drug they are taking for each trial period. The code linking patient to drug sequence was kept in pharmacy and only released after data lock.

Rescue therapy was available if participants did not open their bowels for 3 days during any screening or treatment periods and could not tolerate resulting symptoms. A choice of commonly used medications for constipation was given and participants were given one which they had previously used from prucalopride 2mg tablet, taken orally, senna 7.5mg tablet, 1-4 taken orally and sodium picosulfate 5mg -10mg liquid, taken orally.

If during treatment in either arm, a participant felt they were getting intolerable side effects, first a dose adjustment was permitted (in line with clinical practice) – initially a halving of the dose (this would be done for all tablets at all timepoints) and potentially reduction in frequency (every other day dosing) to a level acceptable. If still unable to tolerate, a subject was advised to discontinue but still advised to complete the diary and return for subsequent visits (this did not exclude the patient from continuation in the trial).

Treatment order was balanced and there were no differences between those that received A or B first (See Table 3.2).

#### Modified PAC-SYM (mPAC-SYM) score

This was calculated using only abdominal pain, discomfort and cramps elements of PAC-SYM since the bowel function element was more precisely measured using the stool diary and the tenesmus and rectal bleeding are more local phenomena less clearly related to colonic function.

### D.1 Supplementary Table S2: Schedule of treatments in randomised cross-over trial of bisacodyl versus buscopan

| *Intervention A = Bisacodyl 10mg once daily; Intervention B = Buscopan 20mg 3 times daily* | | | | | | |
| --- | --- | --- | --- | --- | --- | --- |
| **Visit 5** |  | **Visit 6** |  | **Visit 7** |  | **Visit 8** |
| Start of intervention period 1 | **10 days therapy** | End of intervention  period 1 | Washout | Start of intervention period 2 | **10 days therapy** | End of intervention period 2 |
| *Assignment to* intervention *order* ***AB or BA***  *Baseline measures* | ***Active A & Placebo B*** *or* ***Active B & Placebo A***  *Daily symptom record* | *Final measures* | *At least 7 days* | *Baseline measures* | ***Active A & Placebo B*** *or* ***Active B & Placebo A***  *Daily symptom record* | *Final measures* |

### D. 2 Supplementary Table S3: Demographics of participants by treatment sequence

| *Data shows mean ± SD* | A followed by B (bisacodyl first) n=37 | B followed by A (hyoscine first) n=35 | Significance of difference p |
| --- | --- | --- | --- |
| Age | 44 ± 14 | 43 ± 14 | 0.81 (unpaired t-test) |
| Gender | 35 F 2M | 33 F 2M | 0.95 (Chi-Square) |
| HADS anxiety score | 6.9 ± 4.1 | 7.7 ± 4.7 | 0.47 (unpaired t test) |
| HADS depression score | 4.3 ± 3.7 | 4.6 ± 4.0 | 0.75 (unpaired t test) |
| (m) PAC-SYM | 1.9 ± 0.9 | 1.9 ± 0.8 | 0.74 (unpaired t test) |
| Screening Stool Diary (14-day diary) | | | |
| Total BM Attempts | 20 ± 17 | 17 ± 15 | 0.52 (unpaired t test) |
| Number of CSBM | 1.7 ± 2.9 | 1.7 ± 2.4 | 0.91 (unpaired t test) |
| Average stool consistency (BSFS) ‡ | 2.1 ± 1.3 | 1.8 ± 1.3 | 0.31 (unpaired t test) |

### D3 Statistical analysis plan created before code break

**Part 1**

We will first confirm that the AC MRI motility index (MMI) differs between the FC and IBS-C participants using an unpaired t-test or Mann-Whitney U test depending on the distribution of the data. We will use the same test to compare the FC and IBS-C groups with the control group.

We will then calculate correlations between the manometry parameters and AC and DC MMI separately using the Pearson or Spearman correlation coefficient depending on the distribution of the data; this will be evaluated in all 80 subjects with FC or IBS-C.

We will calculate a lower limit of ‘normal’ motility, defined as <10th centile, for both methods in our healthy volunteer group. We will then calculate a measure of agreement (Cohen’s kappa statistic) between a classification into hypomotile (reduced motility) versus normal / hypermotile (normal or exaggerated motility) [categorised using the 10th centile values from the control group] comparing the gold-standard manometry classification versus one based on either AC or DC MMI in the 80 participants in the FC and IBS-C groups.

We will also calculate overall % agreement and sensitivity / specificity values using the manometry classification as a gold standard.

**Part 2**

We will calculate a correlation coefficient (Pearson or Spearman correlation coefficient as appropriate) between the difference in pain scores between the buscopan and bisacodyl trial periods and the MMI measured in Part 1, to test the hypothesis that there is a positive association between the MMI and pain score changes on buscopan compared with bisacodyl. This will assess whether those with normal / hypermotility experience greater pain relief on Buscopan, while those with hypomotility will do better on bisacodyl.

We will also use linear mixed models to estimate the difference in pain scores comparing buscopan with bisacodyl, to account for the cross-over design, and will include an interaction term between the treatment group and the patients’ MMI to test whether the difference in pain scores between the drugs varies according to the motility index.

We will analyse the difference in pain scores between baseline and trial period for each drug separately (using a Student’s t test if normally distributed or Mann Whitney U test if not) comparing those with motility <10th centile (Group A) with those with normal / hypermotility (Group B) to determine if patients in group A respond

### D4 Sample size considerations

Part 1

Primary objective:

A level of agreement >70% would be sufficiently accurate to allow a choice of the best MRI method and would be in line with the accuracy of most clinical assessments. With a total of 80 participants (with functional constipation (FC) or irritable bowel syndrome with constipation (IBS-C) we could estimate a level of agreement of 70% to within ±10% (95% confidence interval), assuming a proportion of 0.5 in each group (hypomotile versus normal/hypermotile).

A kappa statistic of 0.7 (indicating good agreement) would be estimated to within ±0.16 with 80 participants

Secondary objectives:

Our pilot study data showed an ascending colon MRI motility index mean (SD) of 0.055 (0.044) for functional constipation versus 0.107 (0.07) for constipation-predominant irritable bowel syndrome.

Using these values we calculate we need 27 participants per group to detect such a difference with 90% power with α <0.05 (two-sided). 35 participants per group would be needed to detect a difference of 0.045.

We plan to recruit 40 per group to allow for technical errors in recordings or subject drop-out. We will also recruit 40 volunteers without constipation matched for age and gender with the patient cohort, in order to meet secondary objective 2.

Part 2

There are no previous data on which to base a power calculation. We will ask all patients with FC or IBS-C from study 1 to take part, giving us potentially 80 subjects. Allowing for refusals and dropout we expect a sample size of 60 patients for this study.

This will give us >95% power to detect a correlation coefficient of 0.50 between motility and pain score differences with a significance level of 0.05, indicating that motility accounts for 25% of variance in pain scores which is a reasonable minimal clinically significant difference .

# E: Supplementary Manometry and MRI data Analysis

While every attempt was made to place the manometry catheters so that sensors spanned the rectum, sigmoid and descending colon, invariably in some patients there were too few or no sensors in the rectum or descending colon. However, in all subjects, sensors were present in the sigmoid colon, and therefore this was used the primary site for the manometry analysis. In addition, while all had studies recorded manometry data 2 hours prior to and after the meal, we have found in our previous published work, that the colonic response to a meal is rapid, occurring within a minute of starting to eat. Therefore, in our previous studies (6 ,7) and in this study we have limited the analysis to the 1 hr period prior to and after the meal.

**Manual analysis:** Using our previously developed software (PlotHRM) (8) the manometric traces were examined for the presence of:

1. The Cyclic Motor Pattern (CMP); repetitive propagating pressure events with a cyclic frequency of 1-8/min in either a retrograde or antegrade direction or aligned synchronously across ≥3 sensors. In each trace the start and end time of each episode of the CMP was recorded and then the time occupied by the CMP prior to and after the meal was obtained.

2. High-amplitude propagating contractions (HAPCs); an array of propagating pressure events with at least 2 component pressure waves having a trough- to-peak amplitude of >100 mm Hg. Once identified the following characteristics were obtained, average amplitude of pressure waves, extent, and speed of propagation.

**Automated analysis:** In previous analysis we have shown that pressure waves at frequencies between 1-8cpm dominate in the sigmoid colon of healthy controls, patients with constipation and patients with diarrhoea pre-dominant irritable bowel syndrome(6 ,9). Therefore, here we focused upon pressure waves in this frequency range. The automated analysis was performed using a Bayesian functional mixed-effects model (described in detail elsewhere(7). This approach has been previously used in the analysis of data from patients with slow transit constipation(7) and patients with diarrhoea predominant irritable bowel syndrome (15). The automated approach was broken into 2 distinct sections.

1Dimensional (1D) analysis: Provides an indication of the power of pressure waves (PW) between 1-8 cpm in one hour prior to and after the meal. This analysis did not consider whether the pressure waves are temporally associated with pressure waves in an adjacent channel (i.e. whether the pressure wave is part of a propagating contraction). An increase in the power of PW in response to a meal, indicates that there was an increase in number of PW after a meal. While an increase in the amplitude of the PW may contribute to the increased power, the current analysis approach does not distinguish between these two (count and amplitude) characteristics.

2 Dimensional (2D) analysis: The data used in the 1D analysis were then re-analysed using a 2D group analysis. This analysis provides an indication of the power of propagating pressure waves (PPWs), both antegrade and retrograde, between 1-8 cpm in one hour prior to and after the meal. The 2D analysis assessed the potential temporal relationship between every pressure wave in a channel with pressures waves in the adjacent channel (both the channel above and below). A temporal association between pressure waves in adjacent channels was determined if the duration of the pressure waves in adjacent channels overlapped. If this condition was met the pressure wave formed part of a PPW. PPWs were not defined by distance propagated or the amplitude of the pressure wave. As with the 1D analysis, an increase in the power of PPW in response to a meal, indicates that there was an increase in number of PPW after a meal.

## **E.1 Figure S5: Manometry Trace Example**

Manometry trace example and analysis**:** Automated analysis on a segment of a single manometry recording.


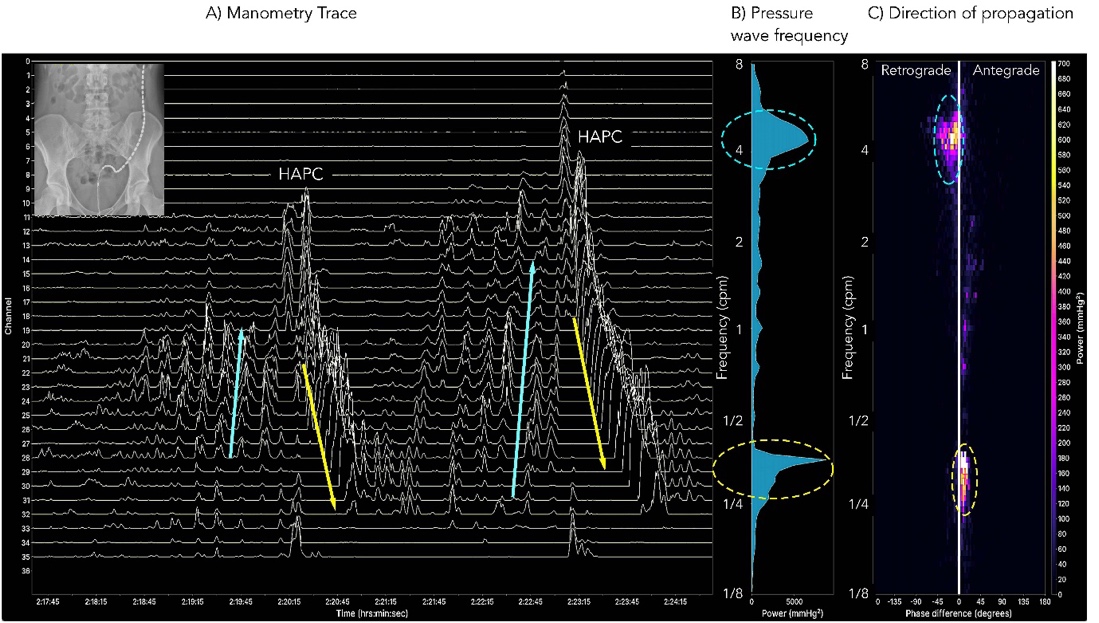
A) shows a section of the manometry trace with 2 HAPCs (yellow arrows) and two clusters of the retrogradely propagating cyclic motor pattern. The X-ray insert shows the location of the catheter for this recording, with sensors in the descending and sigmoid colon).

B) shows the dominant frequencies of pressure waves within the manometry window. A peak can be seen at ~4cpm (the frequency of the cyclic motor pattern) and another at 1/3cpm* (the frequency of the HAPCs).

C) shows the direction of propagation of the dominate frequencies. The 4cpm activity propagates in a retrograde direction (see blue arrows in A) and the 1/3cpm propagates in an antegrade direction (HAPCs, Yellow arrows in A)

* Note that the group analysis in this study focused upon frequencies between 1 – 8cpm. Lower frequencies were excluded.

## E.2 Supplementary MRI Data

### E.2.1 Figure S6 Ascending and Descending Colon Wall Motility

*Figure S6 shows the AC and DC motility index which rose following the meal but as can be seen there was no difference between the groups with wide individual variability (2-way ANOVA, Time effect p<0.0001 for both AC and DC, group effect N Sig AC p=0.8, DC p=0.25) .*

### E.2.2 Figure S7: **Peak volume vs pain on MRI day**

This shows the peak volume in all participants in those with pain (Non-Zero) versus those without pain (Zero values) on the study day. We had planned to correlate the volume of the colon and participants’ reporting of pain, however due to the high number of zero values a direct correlation was not possible. 74 participants reported pain at their peak volume vs 45 who did not.

  
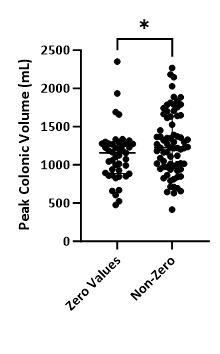


The presence of pain was associated with a significantly higher peak volume 1277(345) versus 1126(410) ml but there was a wide scatter, p=0.04, (unpaired t-test).

### E2.3   Figure S8 Time to Bowel Movement

Compared to healthy volunteers significantly fewer patients opened their bowels within 150 minutes of Moviprep^R^ ingestion after which time they were free to go home.

### E.2.4 Figure S9: Whole gut transit time

Whole gut transit time, as measured by a weighted average position score (WAPS), was significantly longer when all patients were compared to healthy volunteers, with a median (IQR) score of 2.2 (0.6-3.4) and 1 (0-2.3), respectively; p=0.03 (Mann-Whitney)

### E.2.5 Supplementary Table S3: Correlation of baseline Colonic Volumes with other MRI parameters

|  | Baseline Colonic Volume vs.T120 AC Motility | Baseline Colonic Volume vs. Tagging AC T120 | Baseline Colonic Volume vs. Transit Score | Baseline Colonic Volume vs. Time to Bowel Movement |
| --- | --- | --- | --- | --- |
| Spearman R | 0.18 | -0.26 | -0.048 | 0.37 |
| 95 % CI | -0.053 to 0.39 | -0.47 to -0.033 | -0.28 to 0.19 | 0.15 to 0.56 |
| P value | 0.1181 | 0.0217 | 0.6830 | 0.0012 |

### E.2.6 Supplementary Table S4: Pain Scores during Moviprep Challenge

At both Time 60- and 120-minutes post ingestion both patient groups experienced significantly more pain than HVs. FC reported less pain than IBS at 60 minutes, but this difference was lost by 120 minutes.

| Maximum pain scores during Moviprep challenge  Median (range) | | | | | | |
| --- | --- | --- | --- | --- | --- | --- |
| Group | Pain score baseline | Number (%) with >0 score | Pain score T=60 | Number (%) with >0 score | Pain score T=120 | Number (%) with >0 score |
| HV (n=41)​ | 0 (0-0)​ | 2 (5) | 0 (0-0)​ | 7 (17) | 0 (0-0)​ | 9 (22) |
| IBS-C (n= 43)​ | 0.5 (0-1.0)*​ | 25 (58) | 1 (0.5-2.0)*​ † | 35 (81) | 1 (1.0-2.0)*​ | 39 (91) |
| FC (n=36)​ | 0 (0-0.4)*​ | 9 (25) | 0.5 (0-1.0)* | 19 (53) | 1 (0-1.5)*​ | 24 (67) |
| * p<0.05 vs HV, † p<0.05 vs FC | | | | | | |

### **E.2.7 Figure S9: Peak volume vs pain on MRI day**

This shows the peak volume in all participants in those with pain versus those without pain on the study day. We had planned to correlate the volume of the colon and participants’ reporting of pain, however due to the high number of zero values a direct correlation was not possible. 74 participants reported pain at their peak volume vs 45 who did not.

The presence of pain was associated with a significantly higher peak volume 1277(345) versus 1126(410) ml but there was a wide scatter, p=0.04, (unpaired t-test).

### E.2.8 Supplementary Table S5 Agreement between MMI and manometry defined hypomotility.

###

| Table S 6 Number of participants with MMI <10th centile of HVs and / or % time cyclical motility <10^th^ centile of HVs | | | | |
| --- | --- | --- | --- | --- |
|  | N total | Peak MMI <952 | % time cyclical motility <15.1 | Meeting both criteria of hypomotility |
| HV | 35 | 4 | 4 | 0 |
| FC | 25 | 6 | 6 | 1 |
| IBS | 36 | 5 | 5 | 0 |

Only 1 FC patient was hypomotile on both measures. None of the remaining was defined as hypomotile by both measures.

### E2.9 Segmental colonic volumes in patients with enlarged colon versus normal sized colon

| Table S7 Segmental colonic volumes in patients with enlarged colon versus normal sized colon | | | | | |
| --- | --- | --- | --- | --- | --- |
|  | AC | TC | DC | RS | Total |
| Enlarged colon | 323  (348-289) | 399  (495-328) | 195  (252-164) | 145  (225-113) | 1076  (1241-987) |
| Normal sized colon | 251  (260-194) | 228  (315-155) | 86  (120-66) | 106  (140-68) | 703  (793-544) |
| P difference  Mann Whitney test | <0.0001 | <0.0001 | <0.0001 | 0.0004 | <0.0001 |

## E. 3 Manometry Data

### E.3.1 Supplementary Table S8Correlation of Manometry and MRI Measures

| **Correlations of % Time occupied CMPs vs MRI Measures** | CMP vs. Total Baseline Volume | CMP vs. Total T120 Volume | CMP vs. Peak Motility AC | CMP vs. Tagging Max | CMP vs. Transit Score  (WAPS) |
| --- | --- | --- | --- | --- | --- |
| Spearman r | -0.039 | -0.032 | 0.099 | -0.0040 | 0.0088 |
| 95% confidence interval | -0.24 to 0.17 | -0.24 to 0.18 | -0.11 to 0.30 | -0.21 to 0.21 | -0.20 to 0.22 |
|  |  |  |  |  |  |
| P value | 0.7065 | 0.7598 | 0.3394 | 0.9702 | 0.9331 |
|  |  |  |  |  |  |
| Number of XY Pairs | 96 | 95 | 95 | 92 | 94 |

###
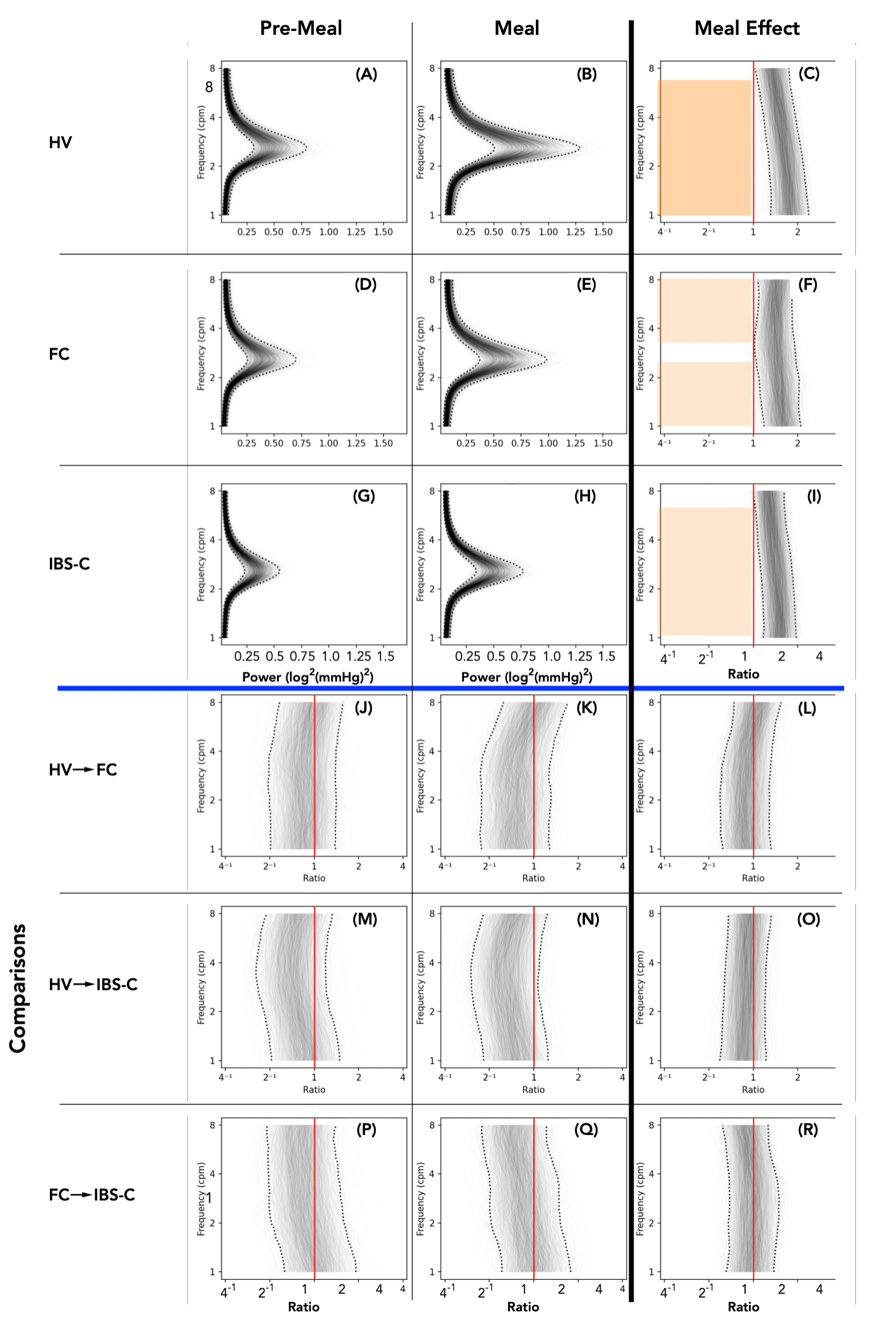
E.3.2 Figure S10: One-dimensional (1D) analysis of pressure waves at frequencies between 1 – 8 cycles per minute (cpm) in the sigmoid colon of patients with a normal diameter colon (top row; a-c), FC patients (middle row; D-F) and IBC-patients (Bottom row; G-I). Baseline data is shown the left column and meal data in the middle colon. In each image, frequency of pressure waves is shown on the Y-axis. In (a, b, d, e, g, h)) power is shown on the X-axis. 2000 overlapping grey lines in each panel represent posterior samples, and the dotted black lines form envelopes of 95% credible intervals. The meal effect for each patient group is shown in c, f & i. When the entire grey envelope lies to one side of the vertical red line (which represents a ratio of 1), this shows a significant deviation (to the left a decrease meal response and to the right of the red line an increase meal response.

Comparisons between the three groups are shown below the solid blue line. The first row compares HV to FC (J & K); the second row HV to IBS-C (M & N) and the final row FC to IBS-C (P & Q). A comparison of the meal effects between groups is shown in the final column (L, O, R). In all images below the blue line the red line at the ratio of 1 lies entirely within the grey envelope, indicating there were no significant differences between groups or between meal effects. Main message of the figure; the meal induced an increase in the power of PW in all three groups, however this increase did not differ between the groups.

**
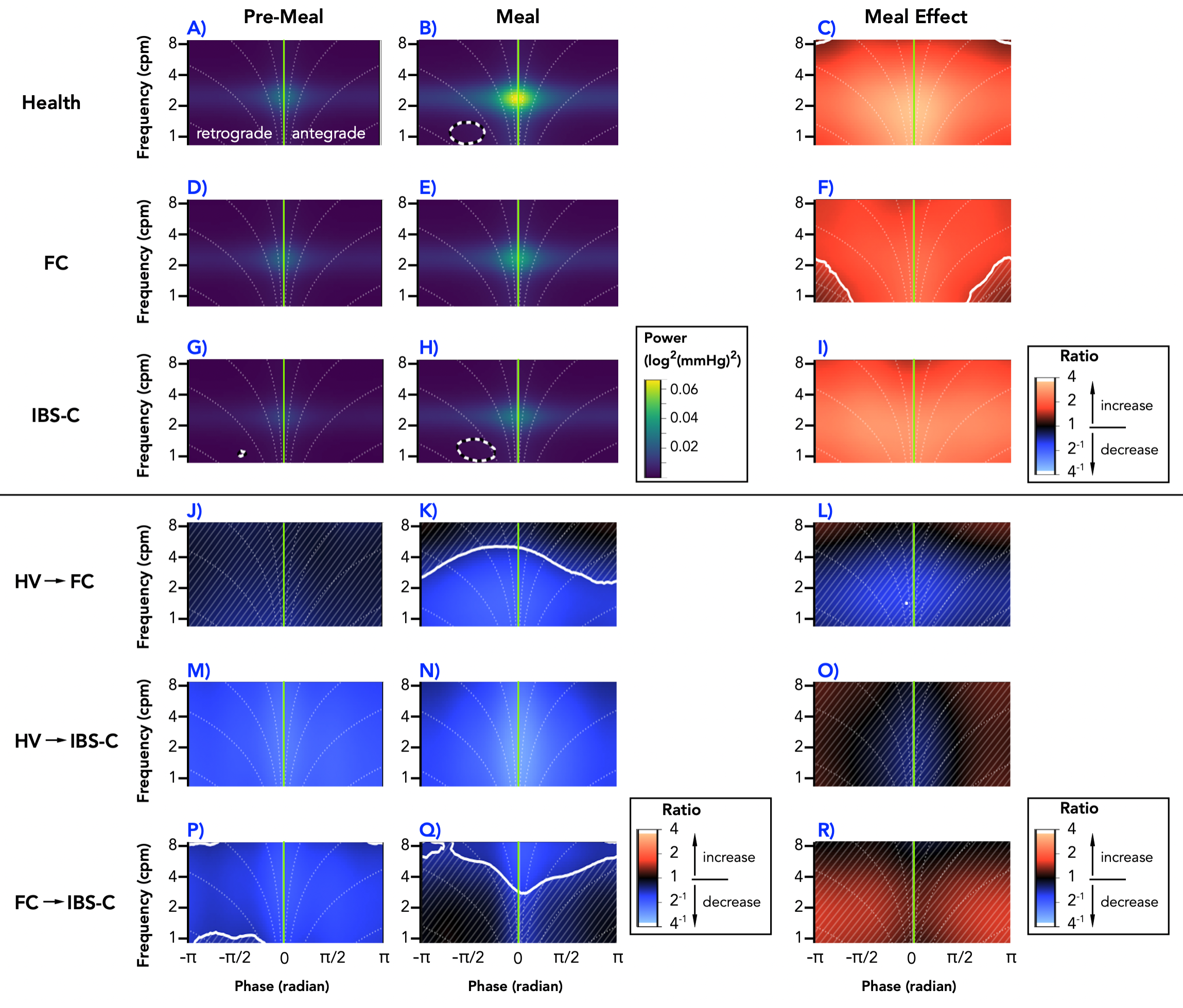
**

### E.3.3 Figure S11: 2-Dimensional analysis of propagating pressure waves (PPW) in the sigmoid colon occurring at frequencies between 1-8cpm. In each panel the vertical line at 0 on the x axis indicates synchronous (non-propagating) activity. Retrograde propagation is to the left of the midline and antegrade propagation to the right. In panels (a, b, d, e, g.h) the green pixels represent increasing power. The first column is baseline data, the second column is data after the meal. Healthy adults are shown in the top row, patients with FC in the second row and those with IBS-C in the 3rd row. The bottom 3 rows (below the solid blackline) compare power across the frequency range between the 3 groups, during premeal (J, M, P) and postmeal (K, N, Q) periods. For each of rows 4 to 6 blue indicates a reduction in PPW power in the second named group compared to the first. For example, in the 4th row HV is named first and FC second. Therefore, blue regions indicate a reduction in power in FC patients compared to HV. In panels J, K, M, N, P, Q, the blue regions demarcated by the solid white line indicate significant reductions. The faint diagonal hatching in these panels indicates regions of non-significance. The meal effect is shown in panels (C-I). The extensive orange/red regions indicates that propagating activity increased in power after the meal. The area demarcated by the solid white lines indicates a significant increase. Comparison of the meal effects between the 3 groups are shown in final panels of the 4th to 6th rows (panels; L, O, R). For each of these panels, blue indicates a reduced meal response in the second group compared to the first. For example, in row 4, the blue in panel (L) indicates the meal effect is FC was reduced compared to HV. However, as the faint diagonal hatching in present throughout the panel this reduction is not significant. The diagonal hatched lines are also present throughout panels O and R, and therefore the meal effect did not differ between any of the groups.

Main message of the Figure: The meal caused a significant increase in the PPWs in all three groups and this meal effect did not differ between the groups (L. O, R). However, during both the baseline and meal periods the PPWs were significantly reduced in IBS-C compared to both HV and patients with FC.

## F Clinical Trial Data

### F. 1 Supplementary Table S 9 Clinical Trial Data by Traditional Subgroup

| Clinical Endpoints | Buscopan​ | Bisacodyl  ​ | Statistical Analysis (Mann-Whitney) ​ |
| --- | --- | --- | --- |
| (IBS n=38 (unpaired – 2 subjects only completed 1 arm)​ | | | |
| Pain  ​ | ​ | | |
| Average worst daily pain  ​ | 1.7 (1.4-2.72)​ | 2.7 (2.1-3.3)​ | p <0.001​ |
| Days with Severe or Very severe Pain ​ | 0 (0-2)​ | 2 (0-4)​ | p <0.001​ |
| Modified (Pain Qs only) PAC-SYM before intervention (range 0-4) ​ | 1.67 (0.92-2.33)​ | 1.67 (1.0-2.0)​ | p = 0.874​ |
| Modified (Pain Qs only) PAC-SYM after intervention (range 0-4) ​ | 1.33 (0.67-2)​ | 2.67 (1.67-3)​ | p <0.001​ |
| Change in PAC-SYM (Post intervention vs Pre)​ | 0 (-1 – 0.33)​ | 1 (0.3-1.67)​ | p <0.001​ |
| Frequency & Consistency​ | | | |
| Weekly average number of bowel movements considered complete (excluding those following rescue) ​ | 0.35 (0-2.1)​ | 1.75 (0-5.95)​ | p = 0.031​ |
| Change in weekly CSBMs vs Screening Period​ | 0 (0-1)​ | 1.15 (0-5.15)​ | p = 0.027​ |
| Weekly average days with constipation (either no bowel movements or at least one type 1-2, or needing rescue) ​ | 4.55 (2.8-7)​ | 1.75 (0-3.5)​ | p <0.001​ |
| Change in weekly average of days with constipation vs screening period ​ | -0.7 (-2.23-0)​ | -3.7 (-5.03- -2.68)​ | p <0.001​ |
| Average stool form over the 10 days (exc BMs following rescue, Bristol Stool Chart Scale 1-7)​ | 2.05 (1.07-3.51)​ | 5.5 (4.9-6)​ | p <0.001​ |

| Clinical Endpoints | Hyoscine  ​ | Bisacodyl  ​ | Statistical Analysis​  (Wilcoxon) ​ |
| --- | --- | --- | --- |
| FC (Paired data n=33)​ | | | |
| Pain  ​ | ​ | | |
| Average worst daily pain  ​ | 1.5 (1-2)​ | 2 (1.6-2.5)​ | p <0.001​​ |
| Days with Severe or Very severe Pain ​ | 0 (0-1)​ | 0.5 (0-1.75)​ | p = 0.026​ |
| Modified (Pain Qs only) PAC-SYM before intervention (range 0-4) ​ | 0.33 (0.17-1.33)​ | 0.67 (0.33-1.17)​ | p = 0.891​ |
| Modified (Pain Qs only) PAC-SYM after intervention (range 0-4) ​ | 0.67 (0-1.67)​ | 1.67 (0.58-2.33)​ | p = 0.005​ |
| Change in PAC-SYM (Post intervention vs Pre)​ | 0 (-0.33 – 0.67)​ | 0.67 (0-1.67)​ | p = 0.019​ |
| Frequency & Consistency​ | | | |
| Weekly average number of bowel movements considered complete (excluding those following rescue) ​ | 0 (0-1.93)​ | 2.8 (0-6.3)​ | p <0.001​​ |
| Change in weekly CSBMs vs Screening Period​ | 0 (-0.55-0.7)​ | 1.5 (0-4.5)​ | p <0.001​​ |
| Weekly average days with constipation (either no bowel movements or at least one type 1-2, or needing rescue) ​ | 5.6 (3.68-6.83)​ | 1.4 (0.7-2.8)​ | p <0.001​ |
| Change in weekly average of days with constipation vs screening period ​ | -0.6 (-2.2-0)​ | -4.1 (-5.7- -2.35)​ | p <0.001​​ |
| Average stool form over the 10 days (exc BMs following rescue, Bristol Stool Chart Scale 1-7)​ | 2.43 (1.46-3.97)​ | 5.19 (4-6.1)​ | p <0.001​​ |

### consort F2 Supplementary Table S10 Effect of enlarged colon on response to hyoscine

| Table S 9 | | | | | |
| --- | --- | --- | --- | --- | --- |
|  | n | Basal mPAC-SYM | Change in mPAC-SYM | Weekly CSBM | Change in CSBM |
| Enlarged colon | 21 | 1.0  (1.0-2.0) | 0.0  (-0.3-0.7)  (-1.0-0.2) | 0.0  (0.0-2.1) | 0.0  (-0.2-1.1) |
| Normal sized colon | 50 | 1.0  (0.3-1.8) | 0.0  (-0.3-0.7) | 0.7  (0.0-2.1) | 0.0  (0.0-0.9) |
| P |  | 0.3‡ | 0.6‡ | 0.5‡ | 0.9‡ |

⭡ t test ‡Mann Whitney test

1. Pritchard SE, Marciani L, Garsed KC, et al. Fasting and postprandial volumes of the undisturbed colon: normal values and changes in diarrhea-predominant irritable bowel syndrome measured using serial MRI. *Neurogastroenterol Motil* 2014;26(1):124-30. doi: 10.1111/nmo.12243 [doi]

2. Menys A, Hamy V, Makanyanga J, et al. Dual registration of abdominal motion for motility assessment in free-breathing data sets acquired using dynamic MRI. *Phys Med Biol* 2014;59(16):4603-19. doi: 10.1088/0031-9155/59/16/4603 [doi]

3. Menys A, Hoad C, Spiller R, et al. Spatio-temporal motility MRI analysis of the stomach and colon. *Neurogastroenterol Motil* 2019;31(5):e13557. doi: 10.1111/nmo.13557 [doi]

4. Pritchard SE, Paul J, Major G, et al. Assessment of motion of colonic contents in the human colon using MRI tagging. *Neurogastroenterol Motil* 2017;29(9) doi: 10.1111/nmo.13091 [doi]

5. Chaddock G, Lam C, Hoad CL, et al. Novel MRI tests of orocecal transit time and whole gut transit time: studies in normal subjects. *Neurogastroenterol Motil* 2014;26(2):205-14. doi: 10.1111/nmo.12249 [doi]

6. Wiklendt L, Mohd Rosli R, Kumar R, et al. Inhibited postprandial retrograde cyclic motor pattern in the distal colon of patients with diarrhea-predominant irritable bowel syndrome. *Am J Physiol Gastrointest Liver Physiol* 2023;325(1):G62-G79. doi: 10.1152/ajpgi.00114.2022 [published Online First: 2023/05/10]

7. Wiklendt L, Costa M, Scott MS, et al. Automated Analysis Using a Bayesian Functional Mixed-Effects Model With Gaussian Process Responses for Wavelet Spectra of Spatiotemporal Colonic Manometry Signals. *Front Physiol* 2020;11:605066. doi: 10.3389/fphys.2020.605066 [published Online First: 2021/03/02]

8. Dinning PG, Wiklendt L, Maslen L, et al. Quantification of in vivo colonic motor patterns in healthy humans before and after a meal revealed by high-resolution fiber-optic manometry. *Neurogastroenterol Motil* 2014;26(10):1443-57. doi: 10.1111/nmo.12408 [doi]

9. Dinning PG, Wiklendt L, Maslen L, et al. Colonic motor abnormalities in slow transit constipation defined by high resolution, fibre-optic manometry. *Neurogastroenterol Motil* 2015;27(3):379-88. doi: 10.1111/nmo.12502 [doi]
